# Simulating Space Conditions Evokes Different DNA Damage Responses in Immature and Mature Cells of the Human Hematopoietic System

**DOI:** 10.3390/ijms241813761

**Published:** 2023-09-06

**Authors:** Leonie Handwerk, Heike Katrin Schreier, Daniela Kraft, Kateryna Shreder, Ruth Hemmersbach, Jens Hauslage, Halvard Bonig, Lisa Wiesmüller, Claudia Fournier, Melanie Rall-Scharpf

**Affiliations:** 1 Department of Biophysics, GSI Helmholtz Center for Heavy Ion Research, 64291 Darmstadt, Germany; 2 Department of Obstetrics and Gynecology, Ulm University, 89075 Ulm, Germany; 3 Department of Gravitational Biology, German Aerospace Center, Institute of Aerospace Medicine, 51147 Cologne, Germany; ruth.hemmersbach@dlr.de (R.H.);; 4 Institute for Transfusion Medicine and Immunohematology, Johann Wolfgang Goethe-University Hospital, and German Red Cross Blood Service, Baden-Wuerttemberg–Hessen, 60528 Frankfurt, Germany

**Keywords:** aging, hematopoietic stem and progenitor cells, leukemia risk, particle irradiation, microgravity, replication stress

## Abstract

The impact of space radiation and microgravity on DNA damage responses has been discussed controversially, largely due to the variety of model systems engaged. Here, we performed side-by-side analyses of human hematopoietic stem/progenitor cells (HSPC) and peripheral blood lymphocytes (PBL) cultivated in a 2D clinostat to simulate microgravity before, during and after photon and particle irradiation. We demonstrate that simulated microgravity (SMG) accelerates the early phase of non-homologous end joining (NHEJ)-mediated repair of simple, X-ray-induced DNA double-strand breaks (DSBs) in PBL, while repair kinetics in HSPC remained unaltered. Repair acceleration was lost with increasing LET of ion exposures, which increases the complexity of DSBs, precluding NHEJ and requiring end resection for successful repair. Such cell-type specific effect of SMG on DSB repair was dependent on the NF-кB pathway pre-activated in PBL but not HSPC. Already under unperturbed growth conditions HSPC and PBL suffered from SMG-induced replication stress associated with accumulation of single-stranded DNA and DSBs, respectively. We conclude that in PBL, SMG-induced DSBs promote repair of radiation-induced damage in an adaptive-like response. HSPC feature SMG-induced single-stranded DNA and FANCD2 foci, i.e., markers of persistent replication stress and senescence that may contribute to a premature decline of the immune system in space.

## 1. Introduction

In light of ongoing efforts towards manned Mars exploration and towards space travel on a regular basis (e.g., ISS, lunar colony), it is of utmost importance to comprehensively understand the risk factors for human health in advance. Astronauts are concomitantly exposed to two unique hazards, namely microgravity and galactic cosmic rays with charged particles of a wide energy range including a high dose contribution by iron ions [1]. Microgravity has been shown to cause degenerative effects on the musculoskeletal, cardiovascular and neurovascular systems [2] as well as immunosuppression due to a decline in lymphocyte numbers and functional impairment of the innate immune system [3]. Particle irradiation in space exerts detrimental effects on the genome integrity aside from the acute effects such as on the retina [4].

Thus, space travel was demonstrated to induce chromosomal aberrations in peripheral blood lymphocytes (PBL) [5], which indeed became a biomarker for space radiation risk [6]. Accumulated epidemiological and experimental evidence also indicates cancer risk for radiation-exposed individuals with leukemia incidence peaking a few decades after exposure [6]. In conclusion, astronauts face an increased risk of leukemia [7,8,9], whereby the impact of combined exposure to particle radiation and microgravity remains to be established.

Chromosomal instabilities underlying leukemia formation range from reciprocal translocations, deletions and insertions to complex structural variations [10,11]. Translocations are driven by classical or alternative non-homologous end joining (NHEJ). These pathways seal double-strand breaks (DSBs) at different sites in the genome, i.e., in an error-prone fashion. Underscoring the leukemogenic role of NHEJ, key factors like DNA-PKcs, LIG4 and PARP1 have frequently been found upregulated in hematologic malignancies [12]. Duplications and deletions are mediated by non-allelic homologous repair of DSBs, complex rearrangements involve template switching at stalled replication forks [13]. In response to photon radiation, NHEJ represents the fast repair component removing the majority of DSBs. Alternative pathways, in particular homology-directed repair mechanisms, require extra time for DNA end resection to promote pairing between the homologies flanking the DSB [14]. Of note, Taucher-Scholz and co-workers demonstrated in human cells that end resection is required to successfully repair complex DSBs such as those generated after exposure to densely ionizing heavy ions [15,16]. In agreement with involvement of slower repair mechanisms, we previously observed prolonged persistence of chromosomal breaks post particle radiation with increasing linear energy transfer (LET) [17].

Due to the complexity of the experimental setup and safety concerns, the effect of microgravity on chromosomal damage induced by radiation during spaceflight has been addressed in two studies only [18,19]. To overcome these limitations, cells were exposed to photon radiation before launching or after landing [20,21,22]. In another approach, radiomimetic was applied during space flight followed by the analysis of DSBs decorated by the modified histone γH2AX [23]. Regardless of the approach, the results either were contradictory or argued against synergy between microgravity and removal of the DNA damage. To create experimental setups for the analysis of multiple replicates, microgravity simulating devices on earth like rotating clinostats for cultured cells have been engineered [2,4]. Most of the studies with primary or immortalized human lymphocytes found evidence for synergy between simulated microgravity (SMG) and photon radiation treatment with regard to various DNA damage responses (DDR). Readouts ranged from apoptosis and chromosome aberrations to γH2AX signals and expression of DNA repair genes [24,25,26,27,28,29]. Conversely, proton and carbon ion treated cells showed no synergy with respect to chromosome aberrations [30] and the opposite effect on apoptosis [31], underscoring the need for systematic analysis of the combined effects of microgravity on photon and heavy ion treated human cells. To this end, we consider models of microgravity on earth valuable tools to investigate such aspects of microgravity, as they facilitate dissection of individual contributions of microgravity, photon and heavy ion (high LET) radiation and combinations thereof as proxy to space radiation.

Hematopoietic stem and progenitor cells (HSPC) represent the origin of leukemia cells [32]. Changes in the differentiation potential of HSPC under microgravity conditions were noticed previously, namely lineage skewing favoring myeloid at the expense of erythroid development [33]. However, DNA repair has not been investigated in human HSPC under SMG. Detailed knowledge of the mechanisms underlying potential dysfunction of DSB repair in space is a first step to identification of molecular targets providing means for prophylactic interventions [12]. In the work presented here, we set out to comparatively analyze DDR and repair in human HSPC and their differentiated progeny, PBL, the human blood cell model prioritized in previous studies [4]. To tackle the technical problem of exposing cells simultaneously to charged particle radiation, such as high LET iron ions, and SMG, we developed a clinostat adapted for accelerator experiments enabling clinorotation of cells in the beam of photons or ions in a preceding study. The 2D fast-rotating clinostat principle was used due to its low shear stress environment for cells in suspension [34].

Employing such equipment enabled studies of the influence of SMG on DDR in HSPC and PBL pre- and post-irradiation. We find basal SMG-driven activation of DDR reminiscent of DNA replication stress in aging cells. This pre-activation is most pronounced in PBL, which also features accelerated repair of photon radiation induced DSBs. By contrast, carbon ion and iron ion induced complex DSBs are repaired at comparable frequencies in HSPC and PBL. Together with in situ analyses of DSB processing, our data suggest that SMG activates NF-кB signaling, favoring fast NHEJ repair in PBL.

## 2. Results

**Figure 1:**
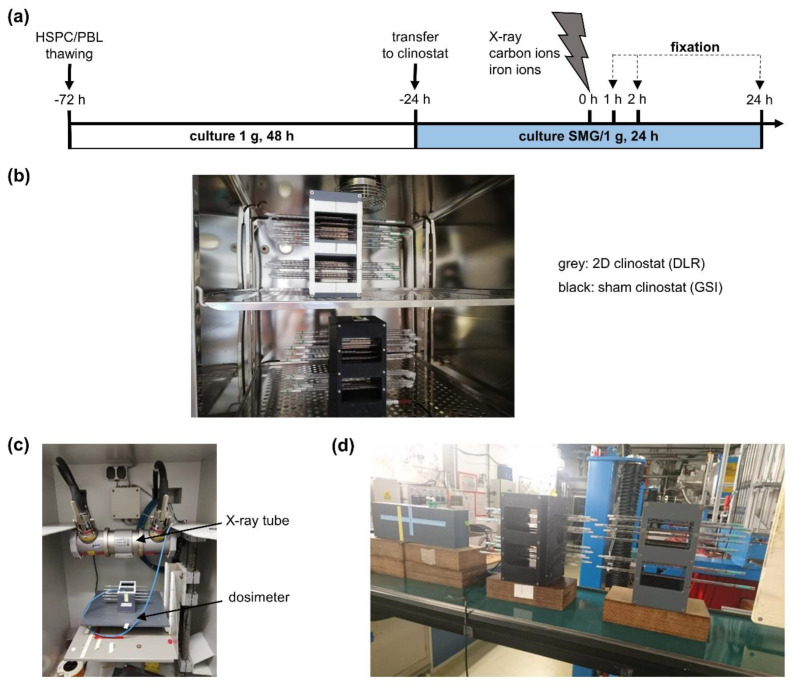
Experimental setup to maintain and irradiate cells in SMG: (**a**) Schematic overview of the experimental set-up for sample preparation for immunofluorescence analysis. HSPC and PBL were thawed 72 h prior to radiation; white bar: culture in a cell culture flask under normal gravity for 48 h; blue bar: culture in a 2D clinostat or sham clinostat under SMG or normal gravity (1 g) conditions, respectively, for 24 h; blizzard: cells were exposed to a dose of 2 Gy X-rays, carbon ions (75 keV/µm) or iron ions (150 keV/µm) at time point 0 h. Cells were then further cultivated, fixed and processed for immunofluorescence analysis at indicated time points. (**b**) 2D Clinostat (grey) with cells rotating in pipettes to simulate microgravity, and sham clinostat (black) with cells in static pipettes. (**c**) X-irradiation unit allowing exposure of cells to X-rays while rotating in the 2D clinostat or sham clinostat. (**d**) Irradiation at heavy ion synchrotron (SIS, GSI).

**Figure 2:**
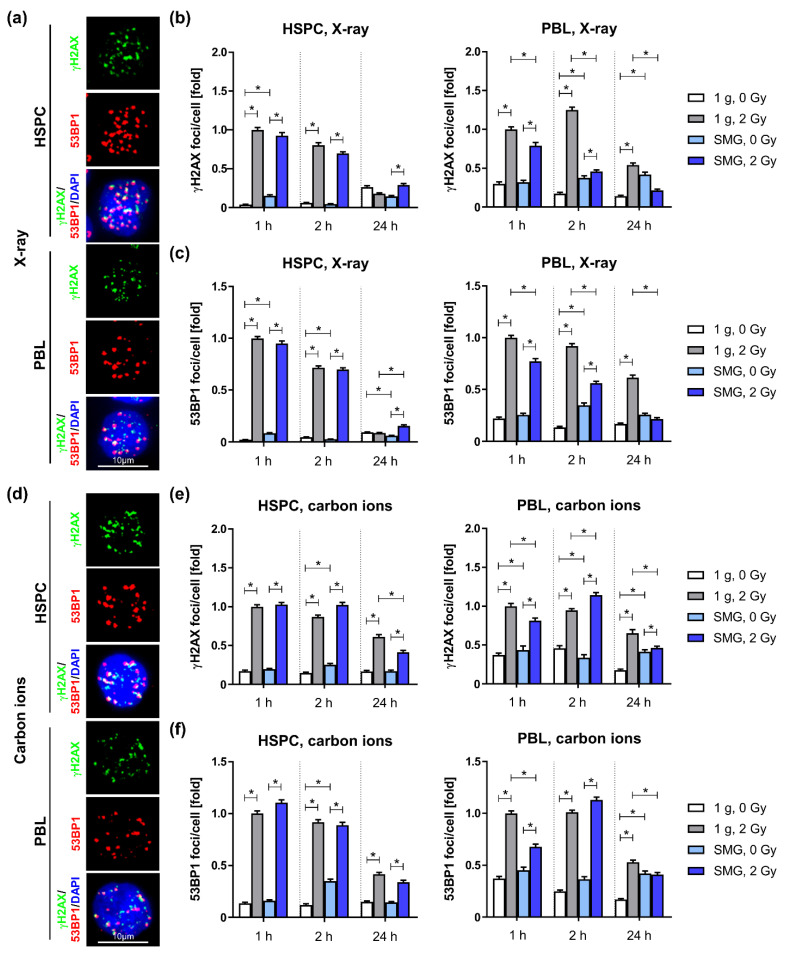
Repair of radiation-induced DNA damage in SMG: HSPC and PBL were cultured under SMG or normal gravity (1 g) conditions for 24 h before they were exposed to a dose of 2 Gy X-rays (**a**–**c**) or carbon ions (75 keV/µm) (**d**–**f**) and further cultivated. At indicated time points cells were fixed, immunolabeled and analyzed by immunofluorescence microscopy. (**a**,**d**) Exemplary immunofluorescence images of nuclei with γH2AX and 53BP1 foci (1 g, 2 Gy, 1 h). X-ray-induced γH2AX (**b**) and 53BP1 foci (**c**). Foci numbers of 68-344 nuclei were scored for each condition and at each time point per experiment. Columns represent relative mean foci numbers; bars, SEM; Mann–Whitney; *, *p* < 0.0001; N = 3. Values were normalized, whereby the values for 1 g, 2 Gy, 1 h were set to 1 for each experiment. 1 relative focus represents the following mean scores for HSPC: γH2AX: 8 foci/cell, 53BP1: 10 foci/cell, PBL: γH2AX: 10 foci/cell, 53BP1: 10 foci/cell. Carbon ion-induced γH2AX (**e**) and 53BP1 foci (**f**). Foci numbers of 30–340 nuclei were scored for each condition and at each timepoint per experiment. Columns represent relative mean foci numbers; bars, SEM; Mann–Whitney; *, *p* < 0.0001; N = 4. One-fold represents the following mean scores for HSPC: γH2AX: 12 foci/cell, 53BP1: 8 foci/cell, PBL: γH2AX: 11 foci/cell, 53BP1: 9 foci/cell.

In order to investigate the influence of SMG on rejoining of radiation-induced DSBs, HSPC and PBL were ex vivo cultured for 48 h before they were transferred to a 2D clinostat and further cultured for 24 h under SMG (Figure 1a,b). Cells were irradiated with a dose of 2 Gy X-rays (Figure 1c), charged carbon (LET 75 keV/μm) or iron particles (LET 150 keV/μm) (Figure 1d), while the cells continued to rotate in the clinostat. Subsequently, the cells were further cultured under SMG or 1 g conditions until they were fixed and processed for immunofluorescence microscopy at indicated time points to monitor the kinetics of γH2AX and 53BP1 nuclear foci (Figure 2 and Figure S1).

### 2.1. Simulated Microgravity Accelerates Repair of Low LET Radiation-Induced DNA Damage in PBL

Exposure to radiation induced a significant increase in γH2AX and 53BP1 nuclear foci in HSPC and PBL under SMG and 1 g conditions. However, in X-ray exposed PBL cultured under SMG, we detected lower numbers of γH2AX and 53BP1 foci numbers at all investigated time points after exposure compared to PBL cultured in 1 g (Figure 2a–c). This suggests that the repair of X-ray-induced DSBs is accelerated in PBL under SMG conditions. By contrast, HSPC cultured under SMG showed slightly, but significantly elevated 53BP1 foci at 24 h post-radiation, but otherwise no differences to 1 g cultured HSPC, pointing to differential regulation of damage repair in PBL and HSPC by SMG.

After exposure to carbon ions, (Figure 2d–f) the effect of SMG was less pronounced in PBL. The numbers of γH2AX and 53BP1 foci were reduced only 1 h and 24 h post-radiation, but slightly increased 2 h after exposure to carbon ions in SMG. HSPC show only minor differences under SMG compared to 1 g conditions in response to carbon ions, visible as a slight reduction of γH2AX foci 24 h post exposure. In contrast to X-ray- and carbon ion-induced DSBs, SMG seems not to accelerate repair of iron ion-induced DSBs (Figure S1). With the exception of 53BP1 foci being decreased in PBL 2 h after exposure, foci numbers in SMG compared to 1 g were comparable or even increased in both cell types. In order to better illustrate changes in repair kinetics we normalized the foci numbers per nucleus of irradiated cells kept in SMG (SMG, 2 Gy) to the mean of the respective 1g controls (1 g, 2 Gy) for each time point (Figure S2). These calculations support an accelerated repair of X-ray-induced DSBs, as normalized mean γH2AX as well as 53BP1 foci noticeably and significantly decline over time, while normalized foci numbers of HSPC even increased (Figure S2a,b). A noticeable decline of the normalized carbon ion-induced foci numbers can only be observed from 2 h to 24 h post radiation in PBL (Figure S2c,d), supporting accelerated repair, but to a lesser extent compared to the repair of X-ray-induced DSBs. Normalized iron ion-induced foci numbers did not indicate changes in repair kinetics (Figure S2e,f).

DSBs induced by low LET radiation (i.e., X-rays, γ-rays) can be relatively well and quickly repaired by NHEJ. In contrast, the repair of damage induced by higher LET charged particles is more challenging for the cells, as the induced DNA lesions are of higher complexity than photon-induced damage [35]. These complex breaks require DNA end resection in all cell cycle phases, leading to repair by homology-directed pathways including HR, single-strand annealing (SSA) and microhomology-mediated end joining (MMEJ) [15,36,37]. In line with this, previous studies suggest high LET-induced damage is not repaired by NHEJ and even inhibits NHEJ mediated repair [37,38]. In light of these findings, it seems plausible that the higher the LET, the more complex are the breaks induced, and the more repair depends on homology-directed pathways. This in mind, the fact that accelerated repair was most pronounced in PBL irradiated with X-rays, but less obvious for intermediate LET carbon ions and undetectable for high LET iron ions, could indicate that SMG promotes or accelerates mainly NHEJ or induces a pathway shift from time-consuming homology-directed repair pathways towards rapid NHEJ.

### 2.2. Simulated Microgravity Does Not Modulate X-ray-Induced Effects on Genomic Integrity, Survival or Differentiation Potential

To assess whether changes in DSB repair activity affect genomic integrity as suggested by others [39,40], we determined micronucleus (MN) frequency in binucleated cells (Figure S3a,b). Using this read out, we did not observe a significant effect of SMG on genomic integrity after exposure to X-rays.

Concomitantly, SMG did not cause apoptosis or necrosis in HSPC or PBL, excluding cell death as a potential confounding factor (Figure S3c–e). Thus, the effects of SMG on the repair of X-ray-induced DSBs observed in PBL did not translate into higher levels of genomic integrity.

To understand whether microgravity affects long-term survival or differentiation processes, we subjected HSPC to colony-forming-unit-assays. To this end, we modified our previous protocol with HSPC seeding in methylcellulose immediately after isolation [41], and embedded HSPC in methylcellulose only after being cultured in the rotating or static clinostat for 24 h (Figure S4a). This modification reduced the total number of colonies by a factor of approximately two yet did not markedly alter the distribution of the different colony types. Under these conditions, exposure to 2 Gy X-rays reduced the survival by a factor of approximately ten irrespective of gravity (Figure S4b). Moreover, irradiation shifted the differentiation capacity of HSPC within the myeloid lineage away from colonies with granulocyte/monocyte (CFU-G/-M/-GM) towards erythroid (BFUe, CFUe) progenitors with less proliferation capacity, i.e., towards smaller (BFUe < 9) and particularly later stage CFUe progenitors (Figure S4c–e) confirming our previously published results [41]. However, in SMG the distribution of the colonies did not change compared to 1 g, both in control and irradiated cells. Therefore, neither the potential of HSPC to grow in the CFU-assay nor the differentiation potential of HSPC was changed after culture in SMG.

### 2.3. Simulated Microgravity Does Not Affect Cell Cycle Progression

**Figure 3:**
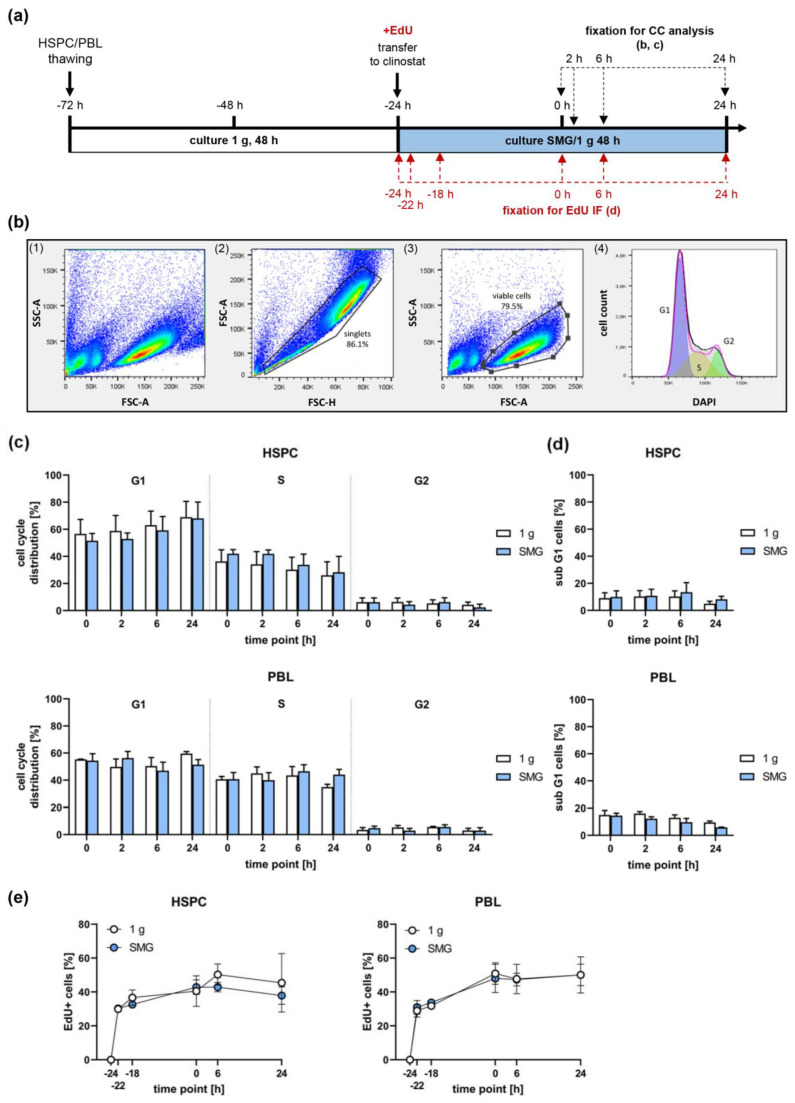
Influence of SMG on proliferation and cell cycle distribution: (**a**) Schematic overview of the cell culture set-up. HSPC and PBL were thawed and cultured in a cell culture flask under normal gravity (1 g) for 48 h (white bar) before they were cultured in the clinostat or sham clinostat under SMG or 1 g conditions, respectively, for up to 48 h (blue bar). (**b**–**d**) Cell cycle and cell death analysis (CC). Cells were fixed at indicated time points and DNA content was analyzed by DAPI staining and flow cytometry. (**b**) Exemplary flow cytometry plots for determining the cell cycle phases. From the starting population (HSPC) (1), the duplicates were excluded by plotting the forward scatter height (FSC-H) versus the forward scatter area (FSC-A) (2). Within the singlets, the population of viable cells was identified by plotting cell size (FSC-A) versus granularity (SSC-A) (3). The cell cycle phases were calculated in viable cells using the Watson Pragmatic algorithm (4). (**c**,**d**) Columns represent the means of the percentages of live cells in G1-, S-, and G2-phase (**c**) and subG1 (**d**) of 3 independent experiments (N); bars, SEM; Wilcoxon test; N = 3, n = 3. (**e**) Ethidium Bromide (EdU)-incorporation. EdU was added when cells were transferred to the clinostat (at −24 h time point). At indicated time points cells were fixed and immunolabeled to analyze EdU incorporation by immunofluorescence (IF) microscopy; Wilcoxon test; N = 3, n = 3.

Since the DSB repair pathway choice is strongly influenced by the cell cycle phase of irradiated cells, we thoroughly investigated cell cycle distribution and S-phase entry of HSPC and PBL during the time frame of our experiments. However, flow cytometry-based analysis of DAPI-stained HSPC and PBL populations cultured in SMG versus 1 g for up to 48 h revealed no significant differences in the cell cycle distribution or cell death detected via the subG1 fraction (Figure 3a–d). In line, HSPC and PBL showed comparable percentages of EdU positive cells when cultured in SMG compared to 1 g (Figure 3e), indicating that SMG did not affect S-phase progression. In conclusion, cell cycle distributions of HSPC and PBL cultured in SMG or 1 g are comparable at the time of radiation exposure (0 h) and investigated time points thereafter. Therefore, it seems that the observed effects of SMG on DSB repair are not caused by changes in cell cycle progression.

### 2.4. Simulated Microgravity Reduces End Processing and Homology-Directed DSB Repair

To further test our hypothesis of SMG-mediated usage of DSB repair mechanisms in PBL, we analyzed the assembly of the proteins RPA and FANCD2, which are key factors for the choice of DSB repair pathways. RPA covers and protects the 3′ single-stranded overhangs generated during end resection, a prerequisite for the search and alignment of homologous sequences and therefore for homology-directed DSB repair pathways [42]. FANCD2 is a key regulator of pathway choices, promoting homologous recombination (HR) in BRCA1/2-proficient and alternative end-joining under BRCA1/2-deficient settings at replication forks [43].

**Figure 4:**
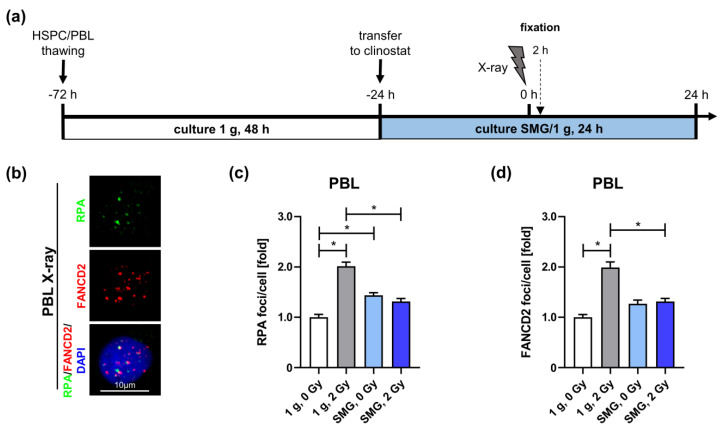
Homology-directed repair of X-ray-induced DNA damage in simulated microgravity (SMG): (**a**) Schematic overview of the experimental set-up for sample preparation for immunofluorescence analysis. PBL were thawed 72 h prior to radiation; white bar: culture in a cell culture flask under normal gravity (1 g) for 48 h; blue bar: culture in clinostat or sham clinostat under SMG or 1 g conditions, respectively, for 24 h; blizzard: cells were exposed to a dose of 2 Gy X-rays at time point 0 h. 2 h post irradiation cells were fixed, immunolabeled and analyzed by immunofluorescence microscopy. (**b**) Exemplary immunofluorescence images of nuclei with RPA and FANCD2 foci (1 g, 2 Gy, 2 h). (**c**) RPA and (**d**) FANCD2 foci. Foci numbers of 36–402 nuclei were scored for each condition per experiment. Columns represent relative mean foci numbers; bars, SEM; Mann–Whitney; *, *p* < 0.0001; N = 3 (PBL). Values were normalized, whereby the values for 1 g, 0 Gy, 2 h were set to 1 for each experiment. One-fold represents the following mean scores: RPA: 15 foci/cell and FANCD2: 15 foci/cell.

Accordingly, we immunodetected focal accumulation of RPA and FANCD2, indicating RPA loading on single-stranded DNA (ssDNA) and FANCD2-activation at DSBs 2 h post-radiation exposure in PBL, cultured in SMG or 1 g (Figure 4). Under 1 g conditions, X-ray exposure led to a significant increase in RPA foci numbers, indicating that DSBs are processed (Figure 4c). In contrast, the lack of such an increase in RPA foci numbers suggested no end resection took place at X-ray-induced DSBs in SMG. Similarly, while X-ray exposure of PBL cultured in 1 g induced FANCD2 foci assembly, FANCD2 foci numbers in irradiated and mock-treated PBL cultured in SMG were comparable (Figure 4d). Exposure to carbon ions even induced a decrease in RPA as well as FANCD2 foci numbers in PBL, and interestingly also in HSPC cultured in SMG (Figure S5). Unchanged or decreased RPA foci numbers in response to radiation in PBL and HSPC cultured in the clinostat suggest that end resection is suppressed in SMG. The corresponding pattern found for FANCD2 foci points to reduced FANCD2-mediated HR. Together, these findings support the idea of preferential usage of NHEJ in SMG. Most interestingly, we noticed that SMG alone increased RPA and FANCD2 foci assembly in PBL as well as HSPC (Figure 4c and Figure S5b,c) suggesting SMG on its own induces endogenous stress.

### 2.5. Simulated Microgravity Induces Replication Stress

**Figure 5:**
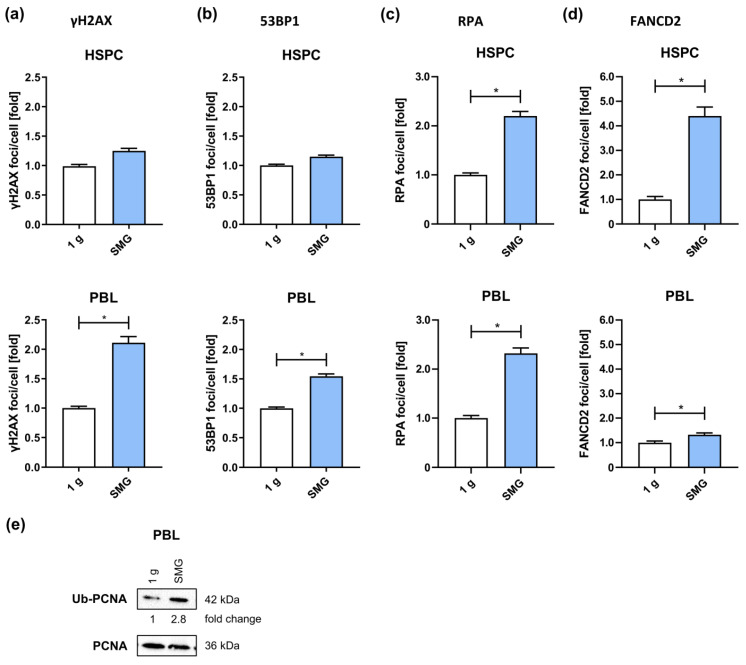
DNA damage and replication stress in SMG: Mean basal (**a**) γH2AX and (**b**) 53BP1 foci numbers of all experiments shown in Figure 2 and Figure S1. HSPC and PBL were cultured under SMG or normal gravity (1 g) conditions for up to 48 h before they were fixed, immunolabeled and analyzed by immunofluorescence microscopy. Foci numbers of all time points (1 h, 2 h, 24 h post mock treatment) were combined. Columns represent relative mean foci numbers; bars, SEM; Mann–Whitney; *, *p* < 0.0001; N = 10 (HSPC); N = 9 (PBL). One-fold represents the following mean scores for HSPC: γH2AX: 1 focus/cell, 53BP1: 1 focus/cell, PBL: γH2AX: 3 foci/cell, 53BP1: 2 foci/cell. Mean basal (**c**) RPA and (**d**) FANCD2 foci numbers of all experiments shown in Figure 4 and Figure S5. HSPC and PBL were cultured under SMG or 1 g conditions for in total 26 h (2 h post radiation exposure) before they were fixed, immunolabeled and analyzed by immunofluorescence microscopy. Columns represent relative mean foci numbers; bars, SEM; Mann–Whitney; *, *p* < 0.0001; N = 2 (HSPC); N = 6 (PBL). Values were normalized, whereby the mean value of all time points for 1 g was set to 1 for each experiment. One-fold represents the following mean scores for HSPC: RPA: 10 foci/cell and FANCD2: 3 foci/cell and PBL: RPA: 6 foci/cell and FANCD2: 4 foci/cell. (**e**) Western Blot showing protein levels of ubiquitinated PCNA and total PCNA in PBL. Protein band intensities of Ub-PCNA were quantified and normalized to PCNA and subsequently to a reference sample to compare band intensities on different blots. Normalized values of PBL cultured at 1 g were set to 1 for each time point. Fold changes of the relative protein levels are indicated below the image; n = 3 (2 h plus 24 h).

Since the higher basal levels of RPA and FANCD2 foci numbers indicated a DNA damage-inducing effect of SMG on its own, we calculated mean values of basal γH2AX, 53BP1, RPA and FANCD2 foci numbers from all the immunofluorescence microscopy experiments at different sampling times during mock-treatments (Figure 2, Figure 4, Figures S1 and S5) to have a more comprehensive look at changes induced by microgravity independently of radiation (Figure 5a–d).

Analysis of basal foci levels in PBL revealed a 2.1-fold and 1.5-fold increase in γH2AX and 53BP1 foci, respectively, in response to SMG, while HSPC showed only a marginal, statistically not significant increase in γH2AX and 53BP1 foci. Thus, microgravity indeed seems to induce DNA damage in PBL but not in HSPC. In contrast, RPA foci numbers were similarly increased (2.2-fold) in HSPC and PBL and FANCD2 foci were increased even 4.4-fold in HSPC, while PBL cultured in SMG showed only a 1.3-fold augmentation. As RPA not only covers ssDNA generated during end resection but also at stalled replication forks—and FANCD2 is a crucial factor to overcome replication fork stalling [44]—we speculated that culture in SMG could induce replication stress that translates into γH2AX- and 53BP1-labeled DNA damage in PBL.

To test this hypothesis, we detected ubiquitination of PCNA, which regulates the choice of DNA damage tolerance pathway to mediate bypass of replication obstacles [45] and thus is a marker for replication stress. Application of an Ub-Lys164-PCNA specific antibody revealed strongly increased Ub-PCNA levels in PBL cultured in SMG for 26 h (24 h + 2 h) and 48 h (24 h + 24 h) compared to 1 g controls (Figure 5e), supporting the induction of severe replication stress by SMG in PBL.

### 2.6. NF-κB Signaling Contributes to the Accelerated Repair of DSBs in PBL Cultured in SMG

We have previously shown that NF-κB is an important regulator of DSB repair, promoting homology-directed repair and NHEJ, and is activated more strongly by DNA damage in PBL than in HSPC [46,47]. As the NF-κB pathway is known to be influenced by true and simulated spaceflight conditions [48], we investigated the involvement of NF-κB in an attempt to understand the molecular causes for the accelerated DSB repair and replication stress in PBL cultured in SMG.

**Figure 6:**
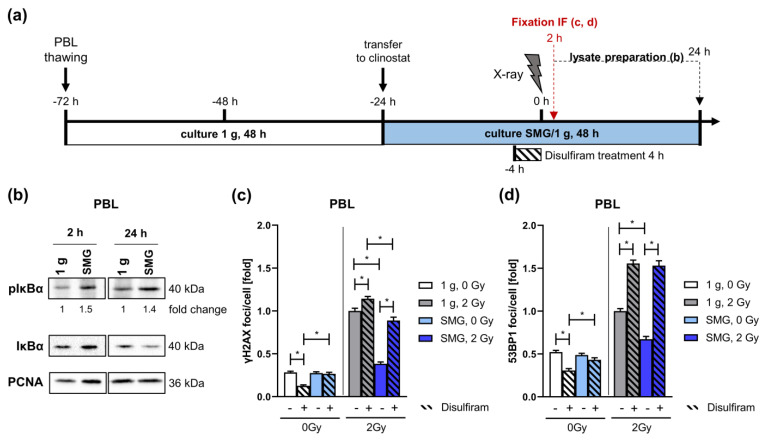
Role of NF-κB signaling in DNA damage repair in SMG: (**a**) Schematic overview of the cell culture set-up. PBL were thawed and cultured in a cell culture flask under normal gravity (1 g) for 48 h (white bar) before they were cultured in the clinostat or sham clinostat under SMG or 1 g conditions, respectively, for 48 h (blue bar). After 20 h (−4 h) cells were treated with the NF-κB-inhibitor Disulfiram (4 μM) or mock-treated with DMSO before they were exposed to a dose of 2 Gy X-rays and further cultivated. 2 h post radiation exposure cells were fixed, immunolabeled and analyzed by immunofluorescence (IF) microscopy. Protein lysates were generated at the 2 h and 24 h time points. (**b**) Representative Western Blots showing protein levels of phosphorylated IκBα and total IκBα. PCNA served as loading control. Protein band intensities were quantified and normalized to loading controls and subsequently to a reference sample to compare band intensities on different blots. Normalized values of PBL cultured at 1 g were set to 1 for each experiment. Mean fold changes of the relative protein levels are indicated below the images; N = 2. γH2AX (**c**) and 53BP1 (**d**) foci. Foci numbers of 105–552 nuclei were scored for each condition per experiment. Columns represent mean foci numbers; bars, SEM; Mann–Whitney; *, *p* < 0.0001; N = 3. 1 g, 2 Gy, DMSO was set to 1. One-fold represents the following mean scores: γH2AX 16 foci/cell and 53BP1: 5 foci/cell.

To confirm that SMG affects activation of NF-κB signaling we performed immunoblotting to determine the phosphorylation status of IκBα (Figure 6a,b). Phosphorylation of IκBα leads to its subsequent degradation and thus NF-κB activation [46]. Increased phospho-IκBα protein levels were detected after culture in the clinostat for 26 h (24 h + 2 h) and 48 h (24 h + 24 h), indicating NF-κB signaling is activated in PBL in response to SMG. Hence, SMG might accelerate removal of X-ray-induced foci by increasing NF-κB activity and thereby promoting DSB repair in PBL.

Consequently, we followed the kinetics of X-ray-induced γH2AX and 53BP1 nuclear foci in PBL cultured in SMG or 1 g in the presence of NF-κB inhibitor Disulfiram [47] (Figure 6a,c,d). As expected, 2 h post-radiation exposure γH2AX and 53BP1 nuclear foci numbers were lower in SMG compared to 1 g. NF-κB inhibition by Disulfiram treatment further increased X-ray-induced foci numbers in 1 g and raised the reduced foci numbers in SMG to the level of PBL in 1 g, namely mock-treated for γH2AX and irradiated for 53BP1. Moreover, Disulfiram treatment reduced radiation-induced RPA foci numbers in PBL cultured in 1 g down to the level of PBL cultured in SMG (Figure S6a), which is in line with the findings that NF-κB signaling promotes end resection [46]. However, Disulfiram did not affect RPA foci in PBL cultured in SMG, excluding a major contribution of end resection and dependent repair pathways to the accelerated DSB repair in irradiated PBL in SMG. FANCD2 foci showed a similar pattern, though with a further decrease in FANCD2 foci also in the presence of Disulfiram, yet at the level of untreated cells (Figure S6b). These results suggest that NF-κB is indeed necessary for the accelerated repair of X-ray-induced DSBs under SMG, whereby end resection does not seem to be involved.

A slight reduction of basal γH2AX and 53BP1 (Figure 6c,d) as well as FANCD2 (Figure S6b) foci numbers by Disulfiram treatment at least in 1 g is compatible with reports showing that under conditions of non-blocking replication stress NF-κB signaling induces low levels of damage due to NF-κB mediated transcription colliding with the replication machinery as well as controlled ROS production as part of a protective adaptation program in primary cells [49,50].

## 3. Discussion

**Figure 7:**
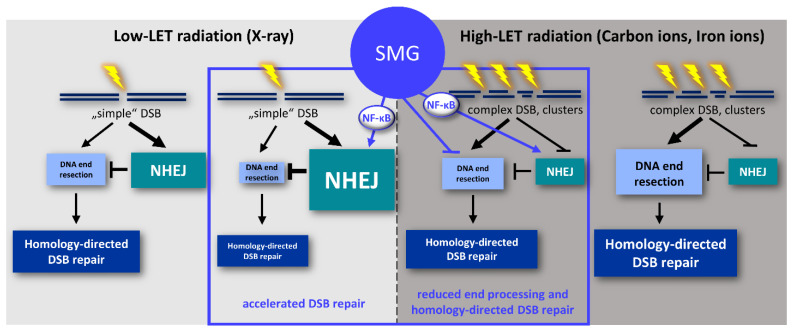
Model of SMG-induced DSB repair activities in PBL. DSB, double-strand break; LET, linear energy transfer; NHEJ, non-homologous end joining.

In light of the divergence of models for space travel used in different studies, each with their different shortcomings, the somewhat contradictory reports on effects of microgravity on DNA repair activities must not surprise [2,4]. Experimental variations range from the source of microgravity, the genotoxic stimulus, organism, cell type, to the chosen functional readout. To the best of our knowledge, our work for the first time investigates DDR in immature and mature cells of the human hematopoietic system after exposure to photon and particle radiation of different LET side-by-side under conditions of 1 g and SMG. The impact of testing HSPC as the origin of radiation-induced leukemogenesis is undoubtedly high [51], with PBL representing the most widely engaged human cell model to investigate effects of radiation and microgravity. We find striking differences in their responses to different radiation qualities of relevance for space travel. While DSB removal in HSPC is largely unaffected by SMG regardless of the radiation source, PBL features accelerated repair of X-ray-induced DSBs. Analysis of molecular switches controlling DSB repair reveals that SMG exploits readily activated NF-κB signaling in PBL to promote fast NHEJ rather than slow but safer end resection-dependent repair pathways. We suggest a model integrating the effects of microgravity and the current knowledge on repair of X-ray and heavy ion-induced DSBs [37] as outlined in Figure 7 and detailed below.

### 3.1. SMG Accelerates Repair of Simple DSBs in PBL

Our previous work established assay conditions for the analysis of radiation responses of HSPC and PBL ex vivo that exclude simple confounding factors such as differences in the cell cycle distribution [47]. Here, these experimental conditions were refined to permit radiation treatment during culture in 1 g and SMG, taking care of uninterrupted rotation in the clinostat during irradiation as well as during transport to and from the radiation equipment. Such details are critical to fully mimic the conditions in space and may also explain differences in the observations made in this work as compared to others. In SMG as compared to 1 g we noticed an accelerated decline of γH2AX- and 53BP1-labeled DSBs in PBL starting within the first 2 h post-γ-ray (2 Gy). Mognato and colleagues [28] observed decelerated disappearance of γH2AX foci yet starting from 6 h post-irradiation (5 Gy). Importantly, these authors exposed PBL to SMG only after irradiation, while in our work cells were kept in SMG for 24 h already before radiation treatment. We purposefully chose such a treatment protocol, as it better resembles conditions during space travel and considers effects requiring continued exposure to SMG such as transcriptional changes. Thus, Singh and colleagues [52] noticed elevated expression of the NHEJ factors Ku70, Ku80 and DNA-PKcs in human promyelocytic leukemic HL-60 cells kept in SMG for 72 h. Differently, Mognato and colleagues [27], again without pre-exposure, did not find differential expression of DSB repair genes in SMG post-γ-ray. Accordingly, we conclude that pre-exposure to SMG promotes DSB repair of photon radiation-induced DSBs in PBL.

NHEJ is well known to account for the rapid removal of the majority of DSBs during the first 2 h after X-ray exposure [53], kinetics prominently seen here in SMG. After irradiation with photons, NHEJ efficiently competes with the more slowly acting DSB repair pathways requiring DNA end resection [54,55]. In support of prevalent NHEJ rather than end-processing pathway usage at X-ray-induced DSBs in SMG-exposed PBL, we noticed absence of RPA foci accumulation above basal levels. Accordingly, nuclear foci of FANCD2, promoting CtIP-dependent end resection and channeling DSB repair to HR rather than NHEJ [56], stayed at basal levels. Charged particle exposure more frequently leads to clustering of DNA damage, particularly for particles with higher LET [57,58] or heavier ions [35,59]. Simpler DSBs, e.g., those induced by X-rays, are reliably repaired by NHEJ, while with increasing complexity due to higher LET of the radiation qualities, repair becomes increasingly dependent on end resection and homology-directed repair [15,53,60]. Complex DSBs show spatiotemporal dynamics of the repair factors NBS1 and 53BP1, indicating a fraction of lesions with delayed recruitment, which first must be processed by base excision repair to generate DSBs [16]. Our analysis of γH2AX foci kinetics revealed that SMG cannot stimulate NHEJ during the fast and early repair phase until 2 h post-irradiation with carbon or iron ions and caused retention of γH2AX until late stages post-treatment for iron ions. Differences in DSB signaling could largely be excluded, as comparable numbers of γH2AX foci were assembled in 1 g and SMG after iron ion exposure. However, under SMG conditions recruitment of the NHEJ factor 53BP1 [61] peaked later post carbon ion exposure than post X-ray (2 h versus 1 h), while RPA as well as FANCD2 foci numbers were reduced rather than augmented as seen in 1 g, suggesting delayed accessibility of DSB repair proteins in SMG. These observations support the idea that repair was accelerated by microgravity in PBL irradiated with X-rays, an effect that was less pronounced with intermediate LET carbon ions and neutralized with high LET iron ions, causing retention of complex DSBs potentially leading to a higher complexity of chromosomal aberrations after ion particle exposure [35,62,63,64,65].

### 3.2. Simulated Microgravity Cell Type Specifically Modulates DSB Repair

Underscoring the attention that needs to be paid to the nature of different biological models investigated in SMG, our side-by-side analysis revealed a radiation response in HSPC that was very different from the one observed in PBL. While PBL showed SMG-accelerated removal of γH2AX/53BP1-labeled damage post X-ray, DSB repair kinetics in HSPC were similar under both 1 g and SMG conditions. Therefore, we conclude that SMG did not stimulate early NHEJ repair of clean DSBs in HSPC. However, during the late repair phase (24 h post X-ray) accumulation of unrepaired DSBs (γH2AX/53BP1) was noticed in HSPC in SMG but not 1 g, suggesting compromised homology-directed repair. Repair of γH2AX-labeled, complex DSBs post iron ion exposure showed a delay in HSPC and PBL in SMG versus 1 g though at different time points (1 h and 24 h, respectively). These observations suggest that DSB repair pathways involving end processing are affected by SMG in HSPC and PBL. In support, RPA foci numbers post carbon ion exposure were comparably affected by SMG in both cell types.

Such cell type-specific impact of microgravity on the repair of radiation-induced DSBs was reminiscent of our earlier findings on higher DSB repair activities in PBL versus HSPC [47]. Thus, augmentation of DSB repair in PBL versus HSPC was more pronounced for NHEJ (22-fold) than the homology-directed repair pathways SSA and HR (on average 9-fold). The NHEJ machinery was found to be more proficient in PBL than HSPC and, as we show here, responsive to further stimulation by microgravity. In our previous work, we demonstrated that elevated NF-кB activity in PBL is required for this activity gap, because DSB removal was antagonized by NF-кB inhibition in PBL but not HSPC. Several other groups (reviewed in [48]) investigated the influence of ionizing radiation and microgravity on NF-кB signaling, mostly reporting NF-кB activation by irradiation. However, even when focusing on human cells, different outcomes were described regarding NF-кB activity in SMG. Yet, similarly as outlined above for space travel models, human cells exposed to microgravity for at least 24 h showed NF-кB activation as a DNA-binding and transcription factor [66,67,68], whereas exposure up to 4 h even caused an opposing effect [69,70,71,72]. Here, we observed augmented NF-кB activity as monitored by phosphorylation of IкBα in PBL, which were cultivated under SMG conditions for 48 h. Strikingly, when treating cells with an inhibitor of the NF-кB pathway, acceleration of DSB removal by SMG was essentially lost (γH2AX/53BP1 foci). Hence, this effect seems to depend on NF-кB activity and is therefore highly cell type specific. We conclude that microgravity stimulates NHEJ specifically in PBL via the pre-activated NF-кB pathway, which must fail in HSPC without active NF-кB [47].

NF-кB was also found to promote homology-directed repair but to a lesser extent [47]. However, SMG neither accelerated repair of complex DSBs generated by iron ions in HSPC nor in PBL. Based on this and our RPA foci data demonstrating failure to accumulate ssDNA post-radiation in SMG, we propose that microgravity antagonizes DNA end resection and therefore also homology-directed repair pathways. Knowing that 53BP1 protects DNA ends from resection [61], it is interesting that 53BP1 foci numbers remained high for 24 h in iron ion exposed PBL particularly under SMG conditions. Prolonged binding of 53BP1 to DNA ends may therefore have contributed to the reduced efficiency of homology-directed repair of complex DSBs in SMG in a futile attempt to perform NHEJ.

While these effects on DSB repair activities did not translate into significant changes in MN formation in our hands, other reports indicate chromosomal translocations detected by FISH after exposure to X-rays and carbon ions [39,40]. Despite the similar nature of their origin, micronuclei and chromosomal translocations are not interchangeable, as they reflect different processing of DNA lesions. Yet, an increase in chromosomal translocations [39,40] is in line with stimulated NHEJ. Lastly, SMG-induced effects on DSB repair activities may cause accumulation of genomic instabilities at sub-microscopic level.

### 3.3. Replication Stress as DSB Repair Pre-Activating Mechanism under SMG Conditions

Clues to differential activation of DSB repair by SMG in HSPC and PBL came from our closer look at basal DDR in SMG. While we neither observed SMG-induced changes in cell death, neither in cell cycle distribution nor in nucleotide incorporation under unperturbed growth conditions, two markers of replication stress were elevated in both HSPC and PBL in SMG as compared to 1 g: RPA, covering ssDNA exposed at uncoupled forks [73], and FANCD2, recruiting HR proteins to reactivate stalled forks [44]. Simultaneously, γH2AX- and 53BP1-foci were upregulated in PBL under SMG conditions, indicating formation of DSBs at stalled replication forks due to incisions [73]. In HSPC, replication stress did not reach the severe outcome of DSBs, which can be explained by more efficient fork remodeling in this cell type in general [74]. Additionally, we observed an 11-fold higher increment of FANCD2 foci numbers under SMG versus 1 g conditions in HSPC compared with PBL suggesting efficient fork protection from nucleases by the Fanconi anemia pathway in the immature cell type [73,75]. Altogether, both cell types showed signs of replication stress, but PBL featured more severe and/or persistent replication stress under SMG conditions, ultimately acquiring DSBs already before radiation treatment.

What can be the reason for the observed stress under unperturbed conditions in SMG? DNA replication barriers are generated by transcription-replication conflicts, DNA secondary structures formed at repetitive elements, nucleotide exhaustion and DNA lesions [76]. Of note, human PBL use mechanisms bypassing barriers fast but error-prone, and the use of these mechanisms is coupled with low level DNA damage as compared with fork remodeling in HSPC [74]. Therefore, PBL are more sensitive to additional, even subtle problems pushing the equilibrium above a threshold triggering DDR. Such an additional stimulus in SMG-exposed PBL may stem from highly active NF-кB-mediated transcription and R-loop formation. In our work, inhibition of NF-кB indeed reduced basal γH2AX/53BP1-damage foci numbers in 1 g but not in SMG, arguing against an isolated NF-кB effect. As a second candidate mechanism, microgravity was reported to inhibit expression of lamin A [77]. Lamin A is known to maintain the positional stability of DDR foci in the nucleus [78], to suppress LINE1 retrotransposons and to guarantee robust DSB repair in concert with the longevity factor SIRT6 [79]. Third, microgravity was demonstrated to activate metabolic pathways [80] and consequently ROS formation [52], which in turn is known to activate NF-кB [81]. Regardless of the mechanism, low-level replication stress in primary cells was shown to activate the PARP1-NF-кB axis and thereby a detoxification program, while above a certain stress threshold, associated with DSBs, DDR involving ATM are induced [50]. These adaptive-like versus canonical stress responses could explain the opposing pattern of RPA/FANCD2 foci alterations pre- and post-radiation in SMG versus 1 g. Most importantly, this mechanism pre-activates DSB repair specifically in PBL with more severe replication stress in SMG and a readily activatable NF-кB pathway, leading to accelerated NHEJ mediated DSB repair in SMG.

## 4. Methods and Materials

### 4.1. Isolation of Primary Hematopoietic Cells

HSPC and PBL were isolated from peripheral blood of healthy donors with the donors’ informed consent. Apheresis products from stem cell donors were provided by the Department of Cellular Therapeutics/Cell Processing of the German Red Cross Blood Service Frankfurt (Frankfurt, Germany; study approval #329/10; #157/10; #155/13). The fraction of CD34+ HSPC was positively selected as described in [62] (CD34 MicroBead Kit, Miltenyi Biotech, Bergisch Gladbach, Germany). Freshly isolated HSPC were subjected to CFU-assay; for all other experimental approaches HSPC from different donors were pooled and frozen in a nitrogen tank (freezing medium: 40% Stem Span SFEM, Stem Cell Technologies Inc., Cologne, Germany; 50% FCS superior, S0615, Sigma-Aldrich Chemie GmbH, Taufkirchen, Germany; 10% DMSO, Applichem, Darmstadt, Germany). With the start of the experiment, cells were thawed in a water bath with a buffer containing PBS + 2% FCS and taken up in expansion media. To obtain actively cycling cell cultures, HSPC were cultured for 48 h in cell culture flasks under standard conditions (37 °C, humidified atmosphere 95%) in serum-free StemSpan SFEM medium supplemented with cytokines: 100 ng/mL Flt-3 ligand (Flt3L), 100 ng/mL Stem Cell Factor (SCF), 20 ng/mL Interleukin-3 (IL3) and 20 ng/mL Interleukin-6 (IL6) (Cytokine Cocktail CC100, both from StemCell Technologies Inc., Vancouver, BC, Canada) under normal gravity conditions (1 g), unless otherwise indicated.

To obtain PBL, peripheral blood was drawn at GSI from healthy volunteers in Vacutainer tubes (BD Vacutainer^®^ blood collection tubes, BD Biosciences, Heidelberg, Germany). PBL were isolated by density-gradient centrifugation which resulted in separation into either plasma or the interphase containing the peripheral blood mononuclear cells (PBMCs). Plasma was collected and heat inactivated (56 °C, 30 min in a water bath). The remaining erythrocytes were removed by hypotonic lysis, as described [62]. To adhere the monocytes, cells were resuspended in X-Vivo-15 medium (Lonza/Biozym Scientific GmbH, Oldendorf, Germany) containing 10% autologous plasma and 100 U/mL Penicillin/100 μg/mL Streptomycin (#P06-07100, PAN Biotech, Aidenbach, Germany) and incubated for 2 h at 37 °C in a humidified atmosphere (95%). PBL in the supernatant were removed and frozen in freezing medium in a nitrogen tank (70% VLE RPMI 1640 Medium, #FG 1415, Biochrom with 2.0 g/L NaHCO_3_ and stable glutamine; very low endotoxin, Biochrom AG/Merck Millipore, Darmstadt, Germany; + 20% FCS superior + 10% DMSO). PBL were thawed in a gentle process in VLE RPMI 1640 Medium + 10% FCS. This was added dropwise. Finally, PBL were cultured in VLE RPMI 1640 Medium (FG 1415, Merck Millipore), supplemented with 20% FCS superior, 10 mM Hepes (#15630-056, Gibco, Darmstadt, Germany) and 100 U/mL Penicillin/100 μg/mL Streptomycin and 2% phytohemagglutinin (PHA.M, Gibco #10576-015, Life Technologies, Carlsbad, CA, USA) at 37 °C in a humidified atmosphere (95%) for 48 h in cell culture flasks under normal gravity conditions (1 g).

### 4.2. Simulated Microgravity Conditions (Clinostat)

Except for Colony-forming-unit-assay, cells were cultured in cell culture flasks for 48 h, before they were split into two fractions: either cells were kept further under normal gravity conditions (1 g), or cells were cultured under SMG at 37 °C in a humidified atmosphere (95%).

Microgravity was simulated using a 2D clinostat (see Figure 1), developed by the German Aerospace Center (DLR, Institute of Aerospace Medicine, Gravitational Biology, Cologne, Germany), enabling the irradiation of suspension cells in SMG. The clinostat was built by using a 3D printing method (fused filament fabrication) with polylactic acid to avoid metal in the clinostat structure to prevent radiation effects after the experiment. The clinostat has 16 slots for 2 mL serological pipettes (Carl Roth, #N236.1, Karlsruhe, Germany). The pipettes in the 2D clinostat rotate around their own axis at a speed of 60 rounds per minute and thus produce a constant change in the gravity vector, preventing the cells from sedimentation [82,83]. This generates simulated microgravity with a maximum residual acceleration of 0.0075 g.

A sham clinostat, also with 16 slots, was designed and built in-house at GSI. In the sham clinostat, the pipettes do not rotate, enabling comparative cultivation of the cells in 2 ml serological pipettes (Sarstedt, #86.1252.001) without SMG (1 g) (static 1 g control). For both systems, 0.8–1 mL of the cell suspension was loaded in the pipettes and thus could be placed in the irradiation window of the clinostat. The pipettes were inserted horizontally into the clinostat and pipettes were sealed with parafilm.

### 4.3. Irradiation with Photons and Heavy Ions and Drug Treatment

To irradiate the cells, they were placed on a lab trolley. A battery was used to keep the power supply on the way from the laboratory to the irradiation site. Cells were exposed to 2 Gy of X-rays using a Seifert Isovolt DSI X-ray tube with a dose rate of ~1Gy/min (16 mA, 250 kV) (Figure 1c). Exposure of cells to 2 Gy of heavy ions was performed at the heavy ion synchrotron (‘Schwerionensynchroton’, SIS, GSI Helmholtz center for heavy ion research, Darmstadt, Germany) (Figure 1d). For irradiation with carbon ions (C_12_, 240 MeV/u), the field size of irradiation was 90 × 60 mm. Samples were irradiated at a depth of 102 mm (Bolus; H_2_O equivalent), with a dose-averaged LET of 75 keV/µm at the position of irradiation (modulator from Ullrich Weber, GSI, was used). The same setup was calculated for iron ions (Fe_56_, 1 GeV/u) irradiation resulting in a LET of 150 keV/µm. After irradiation, cells were either cultivated or fixed for the different experimental endpoints (again under SMG conditions or 1 g). To inhibit NF-κB, cells were treated with 4 µM Disulfiram or mock treated 4 h prior to radiation exposure.

### 4.4. Quantitative Immunofluorescence Microscopy

At indicated timepoints post-irradiation, cells were fixed in 3.7% formaldehyde (VWR International, Bruchsal, Germany) for 10 min. For immunofluorescence analysis of RPA and FANCD2 foci, cells were additionally pre-extracted in cold pre-extraction buffer (20 mM HEPES, pH 7.4; 50 mM NaCl; 1 mM EDTA; 3 mM MgCl_2_; 300 mM Sucrose; 0.5% Triton X-100) for 1 min prior to fixation. Cells were then cytospinned on poly-L-Lysine (Sigma) covered glass slides and fixed again in 3.7% formaldehyde for 10 min. For immunofluorescence staining, fixed slides from all time points were collected, washed 3 times for 5 min in PBS and permeabilized with 0.5% Triton for 10 min. To avoid unspecific binding, slides were blocked in 5% goat serum for 1 h at RT, followed by immunostaining with primary antibodies, anti-53BP1 (rabbit, polyclonal, NB100-304, Novus Biologicals, Littleton, CO, USA) and anti-phospho-histone H2A.X (Ser139, mouse, monoclonal, JBW301, Millipore) or anti-FANCD2 (rabbit, monoclonal, EPR2302, Abcam, Cambridge, UK) and anti-RPA (mouse, monoclonal, Ab-2, RPA34-19, Calbiochem, San Diego, CA, USA) diluted in 5% goat serum, at 37 °C for 1 h or at 4 °C overnight. After another washing step (3 × 5 min in PBS) slides were incubated with secondary antibody Alexa Fluor555-anti-mouse, Alexa Fluor488-anti-mouse or Alexa Fluor555-anti-rabbit (Invitrogen, Waltham, MA, USA) 1 h at 37 °C. Final washing (3 × 5 min in cold 0.1% triton) was performed, before slides were mounted in Vectashield containing DAPI (Vector Laboratories, Newark, CA, USA) and sealed under cover slips. Nuclear immunofluorescence signals were imaged with a BZ-9000 microscope (Keyence, Singapore) or inverted Axio Observer 7 microscope (Zeiss, Oberkochen, Germany) using a 100 × objective. Automated identification and quantification of foci was performed using Cell Profiler software [84].

### 4.5. Micronucleus Test

After preculture for 48 h, cells were irradiated with 2 Gy X-rays (+untreated control), immediately treated with 4.5 μg/mL cytochalasin B (Sigma) and then half of the irradiated and half of the non-irradiated samples were transferred to the 2D clinostat. The remaining samples were transferred to the sham clinostat. Then, 48 h later, the cells were harvested and prepared for micronucleus analysis by fixation with methanol/glacial acetic acid (3:1) for preservation. The cells did not require hypotonic treatment for slide preparation, thus making it possible to preserve the morphology of both necrotic and apoptotic cells [85]. The micronuclei were visualized by Giemsa staining. Therefore, the slides were immersed in a Giemsa solution (1:10) diluted with Sörensen buffer (pH 6.8) and incubated for 20 min. They were then soaked in Sörensen buffer for 2 min and washed with deionized water. To protect the slides from further contamination and color fading, they were mounted with Eukitt and covered with a cover slip. The slides were imaged using an Echo Revolve microscope (Echo, software version 13.3.1). Next, 500 cells were counted per slide. Binuclear cells and micronuclei in binuclear cells were counted. Apoptotic cells have been identified based on condensed or fragmented DNA. Necrotic cells have been identified based on a glassy and vacuolated morphology, as during necrosis organelles are swelling, the plasma membrane is ruptured.

### 4.6. Colony-Forming-Unit-Assay

CD34+ HSPC were cultured for 24 h in 2 mL serological pipettes, either under normal conditions (1 g) or under SMG. Afterwards, cells were exposed to 2 Gy of X-rays; unirradiated cells served as controls. As described elsewhere [41], cells were embedded in semisolid methycellulose plus cytokines (Methocult GF H4434, #04434, StemCell technologies) and kept at 37 °C in a humidified atmosphere (95%), which allows cells to differentiate within 14 days into cells of the myeloid lineage. Colonies were counted and identified based on morphological pattern using light microscopy. The multi-potential and immature CFU-GEMM progenitors were recognized due to the presence of erythroid and other cells (granulocytes, macrophages, megakaryocytes) within one colony. Colonies with clusters of predominantly hemoglobinized erythroblasts cells were classified as erythroid progenitors: primitive burst-forming unit-erythroides with high proliferative capacity (BFU-E, >3 clusters) and later-stage colony-forming unit-erythroid (CFU-E, 1–2 clusters). Colonies containing granulocytes (-G), macrophages (-M) or a combination of both (-GM) were scored and grouped as CFU-G/-M/-GM.

### 4.7. Cell Cycle and Sub G1 Analysis

The distribution of the cells in the different cell cycle phases was determined by measuring the DNA content of DAPI stained cells by flow cytometry (BD FACS Canto with FACS Diva software version 4.1). To this end, cells were fixed in 3.7% PFA for 45 min at indicated time points and incubated with 1 μg/mL DAPI for 30 min in the dark. Data analysis was conducted using FLOWJO software. Thereby, cell cycle analysis was performed using Waston’s Progmatic algorithm within the FLOWJO software. In addition, the percentage of apoptotic cells was determined by the sub-G1 method.

### 4.8. EdU Staining

After preculture for 48 h, the cells were treated with 1 µM EdU (EdU Click FC ROTI^®^kit, Carl Roth, Karlsruhe, Germany). Then, one half of the samples was transferred to the clinostat, the other half to the sham clinostat (1 g).

At indicated time points, the cells were harvested, washed with PBS and fixed with 3.7% formaldehyde (RT, 15 min in the dark). Cells were washed with 1 mL PBS + 3% BSA and the cell membranes permeabilized with PBS + 0.5% Triton (RT, 20 min). After another washing step, the fluorescent dye and the reaction cocktail was added (30 min in the dark). Finally, the cells were mounted in eukit and covered with a cover slip. The slides were imaged using an Echo Revolve microscope (Echo, software version 13.3.1). 1000 cells were counted per slide and the percentage of EdU+ cells was determined.

### 4.9. Immunoblotting

Cellular lysates were prepared and analyzed by Western Blotting as previously described [86]. Proteins were extracted by incubating the cells in lysis buffer (50 mM Tris, pH 7.4; 150 mM NaCl; 2 mM EGTA; 2 mM EDTA; 25 mM NaF; 25 mM β-glycerophosphate; 0.1 mM NaV; 0.2% Triton X-100; 0.3% Nonidet P40; proteinase inhibitor, Roche, Basel, Switzerland). Following centrifugation, protein concentrations of supernatants were determined by the BCA™ Protein Assay Kit (Thermo Scientific, Waltham, MA, USA). Next, 30–50 μg of protein per sample was separated electrophoretically using 10% SDS–PAGE gels and blotted onto Hybond™-C-Extra Nitrocellulose membranes (GE Healthcare, Chicago, IL, USA). Proteins of interest were detected using the following antibodies: anti-phospho-IkBα (Ser32/36, mouse, monoclonal, 5A5, Cell Signalling, Danvers, MA, USA), anti-IkBα (rabbit, polyclonal, C-21, Santa Cruz, Singapore), anti-PCNA (mouse, monoclonal, PC10, Abcam), and anti-ubiquityl-PCNA (Lys164, rabbit, monoclonal, D5C7P, Cell Signaling). Chemiluminescence signals were detected on a ChemiDocMP System (BioRad, Hercules, CA, USA) using Clarity^TM^ Western ECL Substrate (BioRad) and Band intensities were quantified using ImageLab software 4.1. Intensity values of the protein of interest were normalized to the values of the corresponding loading control.

### 4.10. Statistics

Statistical analyses were performed using GraphPad Prism 9.0 software. The Mann–Whitney two-tailed test or Wilcoxon test were applied for pairwise comparisons. Significance levels were not adjusted for multiple comparisons. Differences were considered as significant for *p* values < 0.0001 in immunofluorescence experiments, otherwise for *p* values < 0.05.

## 5. Conclusions

Our work reveals that clinorotation (simulated microgravity) induces replication stress and resulting DNA damage in immature human hematopoietic cells and their differentiated counterparts, in particular. In differentiated cells SMG significantly accelerated NF-кB-dependent repair of radiation-induced DSBs with low complexity. Such DSB repair favors error-prone NHEJ, which has been associated with leukemic genome mutations [12]. Since these SMG-induced changes were seen primarily in mature PBL rather than in HSPC which are the cells of origin of myeloid leukemias, NHEJ is apparently not a major risk of microgravity in space. On the other hand, HSPC accumulated FANCD2 signals more prominently than PBL. FANCD2 activation is a marker of persistent replication stress at the onset of oncogene-induced senescence [87,88]. Intriguingly, it is known that both replication stress and radiation exposure of HSPC accelerate premature aging [89,90]. Our approach to expose samples in parallel to SMG and radiation give us clear indications of what might happen in space and help to identify involved pathways and targets. Nevertheless, a simulation needs a final verification under real spaceflight and microgravity conditions. In the light of these and earlier observations suggesting attenuation of the immune system by space travel [3,91,92], it remains important to monitor the health status of astronauts to fully understand potential long-term effects of space travel.

## Data Availability

All data included in this paper are available upon reasonable request to the corresponding author.

## Supplementary Materials

The following supporting information can be downloaded at https://www.mdpi.com/article/10.3390/ijms241813761/s1.

## References

[1] Durante M., Cucinotta F.A. (2011). Physical Basis of Radiation Protection in Space Travel. Rev. Mod. Phys..

[2] Yatagai F., Honma M., Dohmae N., Ishioka N. (2019). Biological Effects of Space Environmental Factors: A Possible Interaction between Space Radiation and Microgravity. Life Sci. Sp. Res..

[3] Guéguinou N., Huin-Schohn C., Bascove M., Bueb J.-L., Tschirhart E., Legrand-Frossi C., Frippiat J.-P. (2009). Could Spaceflight-Associated Immune System Weakening Preclude the Expansion of Human Presence beyond Earth’s Orbit?. J. Leukoc. Biol..

[4] Moreno-Villanueva M., Wong M., Lu T., Zhang Y., Wu H. (2017). Interplay of Space Radiation and Microgravity in DNA Damage and DNA Damage Response. NPJ Microgravity.

[5] George K., Chappell L.J., Cucinotta F.A. (2010). Persistence of Space Radiation Induced Cytogenetic Damage in the Blood Lymphocytes of Astronauts. Mutat. Res.—Genet. Toxicol. Environ. Mutagen..

[6] Ali Y.F., Cucinotta F.A., Ning-Ang L., Zhou G. (2020). Cancer Risk of Low Dose Ionizing Radiation. Front. Phys..

[7] Durante M., Cucinotta F.A. (2008). Heavy Ion Carcinogenesis and Human Space Exploration. Nat. Rev. Cancer.

[8] Cucinotta F.A. (2014). Space Radiation Risks for Astronauts on Multiple International Space Station Missions. PLoS ONE.

[9] Cucinotta F.A., To K., Cacao E. (2017). Predictions of Space Radiation Fatality Risk for Exploration Missions. Life Sci. Sp. Res..

[10] Downing J.R., Wilson R.K., Zhang J., Mardis E.R., Pui C.-H., Ding L., Ley T.J., Evans W.E. (2012). The Pediatric Cancer Genome Project. Nat. Genet..

[11] Meyer C., Larghero P., Almeida Lopes B., Burmeister T., Gröger D., Sutton R., Venn N.C., Cazzaniga G., Corral Abascal L., Tsaur G. (2023). The KMT2A Recombinome of Acute Leukemias in 2023. Leukemia.

[12] Valikhani M., Rahimian E., Ahmadi S.E., Chegeni R., Safa M. (2021). Involvement of Classic and Alternative Non-Homologous End Joining Pathways in Hematologic Malignancies: Targeting Strategies for Treatment. Exp. Hematol. Oncol..

[13] Zhang F., Gu W., Hurles M.E., Lupski J.R. (2009). Copy Number Variation in Human Health, Disease, and Evolution. Annu. Rev. Genom. Hum. Genet..

[14] Jeggo P.A., Geuting V., Löbrich M. (2011). The Role of Homologous Recombination in Radiation-Induced Double-Strand Break Repair. Radiother. Oncol..

[15] Averbeck N.B., Ringel O., Herrlitz M., Jakob B., Durante M., Taucher-Scholz G. (2014). DNA End Resection Is Needed for the Repair of Complex Lesions in G1-Phase Human Cells. Cell Cycle.

[16] Jakob B., Dubiak-Szepietowska M., Janiel E., Schmidt A., Durante M., Taucher-Scholz G. (2020). Differential Repair Protein Recruitment at Sites of Clustered and Isolated DNA Double-Strand Breaks Produced by High-Energy Heavy Ions. Sci. Rep..

[17] Rall M., Kraft D., Volcic M., Cucu A., Nasonova E., Taucher-Scholz G., Bönig H., Wiesmüller L., Fournier C. (2015). Impact of Charged Particle Exposure on Homologous DNA Double-Strand Break Repair in Human Blood-Derived Cells. Front. Oncol..

[18] Bender M.A., Gooch P.C., Kondo S. (1967). The Gemini-3 S-4 Spaceflight-Radiation Interaction Experiment. Radiat. Res..

[19] Bender M.A., Gooch P.C., Kondo S. (1968). The Gemini XI S-4 Spaceflight-Radiation Interaction Experiment: The Human Blood Experiment. Radiat. Res..

[20] Horneck G. (1999). Impact of Microgravity on Radiobiological Processes and Efficiency of DNA Repair. Mutat. Res.—Fundam. Mol. Mech. Mutagen..

[21] Wu H., George K., Willingham V., Cucinotta F.A. (2001). Comparison of Chromosome Aberration Frequencies in Pre- and Post-Flight Astronaut Lymphocytes Irradiated in Vitro with Gamma Rays. Phys. Med..

[22] Greco O., Durante M., Gialanella G., Grossi G., Pugliese M., Scampoli P., Snigiryova G., Obe G. (2003). Biological Dosimetry in Russian and Italian Astronauts. Adv. Sp. Res..

[23] Lu T., Zhang Y., Kidane Y., Feiveson A., Stodieck L., Karouia F., Ramesh G., Rohde L., Wu H. (2017). Cellular Responses and Gene Expression Profile Changes Due to Bleomycin-Induced DNA Damage in Human Fibroblasts in Space. PLoS ONE.

[24] Risin D., Pellis N.R. (2001). Modeled Microgravity Inhibits Apoptosis in Peripheral Blood Lymphocytes1. Vitr. Cell. Dev. Biol.—Anim..

[25] Mosesso P., Schuber M., Seibt D., Schmitz C., Fiore M., Schinoppi A., Penna S., Palitti F. (2001). X-Ray-Induced Chromosome Aberrations in Human Lymphocytes in Vitro Are Potentiated under Simulated Microgravity Conditions (Clinostat). Phys. Med..

[26] Canova S., Fiorasi F., Mognato M., Grifalconi M., Reddi E., Russo A., Celotti L. (2005). “Modeled Microgravity” Affects Cell Response to Ionizing Radiation and Increases Genomic Damage. Radiat. Res..

[27] Mognato M., Celotti L. (2005). Modeled Microgravity Affects Cell Survival and HPRT Mutant Frequency, but Not the Expression of DNA Repair Genes in Human Lymphocytes Irradiated with Ionising Radiation. Mutat. Res..

[28] Mognato M., Girardi C., Fabris S., Celotti L. (2009). DNA Repair in Modeled Microgravity: Double Strand Break Rejoining Activity in Human Lymphocytes Irradiated with γ-Rays. Mutat. Res.—Fundam. Mol. Mech. Mutagen..

[29] Kumari R., Singh K.P., DuMond J.W. (2009). Simulated Microgravity Decreases DNA Repair Capacity and Induces DNA Damage in Human Lymphocytes. J. Cell. Biochem..

[30] Manti L., Durante M., Cirrone G., Grossi G., Lattuada M., Pugliese M., Sabini M., Scampoli P., Valastro L., Gialanella G. (2005). Modelled Microgravity Does Not Modify the Yield of Chromosome Aberrations Induced by High-Energy Protons in Human Lymphocytes. Int. J. Radiat. Biol..

[31] Dang B., Yang Y., Zhang E., Li W., Mi X., Meng Y., Yan S., Wang Z., Wei W., Shao C. (2014). Simulated Microgravity Increases Heavy Ion Radiation-Induced Apoptosis in Human B Lymphoblasts. Life Sci..

[32] Tothova Z., Krill-Burger J.M., Popova K.D., Landers C.C., Sievers Q.L., Yudovich D., Belizaire R., Aster J.C., Morgan E.A., Tsherniak A. (2017). Multiplex CRISPR/Cas9-Based Genome Editing in Human Hematopoietic Stem Cells Models Clonal Hematopoiesis and Myeloid Neoplasia. Cell Stem Cell.

[33] Plett P.A., Abonour R., Frankovitz S.M., Orschell C.M. (2004). Impact of Modeled Microgravity on Migration, Differentiation, and Cell Cycle Control of Primitive Human Hematopoietic Progenitor Cells. Exp. Hematol..

[34] Hauslage J., Cevik V., Hemmersbach R. (2017). Pyrocystis Noctiluca Represents an Excellent Bioassay for Shear Forces Induced in Ground-Based Microgravity Simulators (Clinostat and Random Positioning Machine). NPJ Microgravity.

[35] Asaithamby A., Chen D.J. (2011). Mechanism of Cluster DNA Damage Repair in Response to High-Atomic Number and Energy Particles Radiation. Mutat. Res. Mol. Mech. Mutagen..

[36] Davis A.J., Chen D.J. (2014). Complex DSBs: A Need for Resection. Cell Cycle.

[37] Mladenova V., Mladenov E., Stuschke M., Iliakis G. (2022). DNA Damage Clustering after Ionizing Radiation and Consequences in the Processing of Chromatin Breaks. Molecules.

[38] Wang H., Zhang X., Wang P., Yu X., Essers J., Chen D., Kanaar R., Takeda S., Wang Y. (2010). Characteristics of DNA-Binding Proteins Determine the Biological Sensitivity to High-Linear Energy Transfer Radiation. Nucleic Acids Res..

[39] Hada M., Ikeda H., Rhone J.R., Beitman A.J., Plante I., Souda H., Yoshida Y., Held K.D., Fujiwara K., Saganti P.B. (2019). Increased Chromosome Aberrations in Cells Exposed Simultaneously to Simulated Microgravity and Radiation. Int. J. Mol. Sci..

[40] Yamanouchi S., Rhone J., Mao J.H., Fujiwara K., Saganti P.B., Takahashi A., Hada M. (2020). Simultaneous Exposure of Cultured Human Lymphoblastic Cells to Simulated Microgravity and Radiation Increases Chromosome Aberrations. Life.

[41] Kraft D., Ritter S., Durante M., Seifried E., Fournier C., Tonn T. (2015). Transmission of Clonal Chromosomal Abnormalities in Human Hematopoietic Stem and Progenitor Cells Surviving Radiation Exposure. Mutat. Res..

[42] Symington L.S. (2016). Mechanism and Regulation of DNA End Resection in Eukaryotes. Crit. Rev. Biochem. Mol. Biol..

[43] Kais Z., Rondinelli B., Holmes A., O’Leary C., Kozono D., D’Andrea A.D., Ceccaldi R. (2016). FANCD2 Maintains Fork Stability in BRCA1/2-Deficient Tumors and Promotes Alternative End-Joining DNA Repair. Cell Rep..

[44] Raghunandan M., Chaudhury I., Kelich S.L., Hanenberg H., Sobeck A. (2015). FANCD2, FANCJ and BRCA2 Cooperate to Promote Replication Fork Recovery Independently of the Fanconi Anemia Core Complex. Cell Cycle.

[45] Kanao R., Masutani C. (2017). Regulation of DNA Damage Tolerance in Mammalian Cells by Post-Translational Modifications of PCNA. Mutat. Res..

[46] Volcic M., Karl S., Baumann B., Salles D., Daniel P., Fulda S., Wiesmuller L. (2012). NF-KappaB Regulates DNA Double-Strand Break Repair in Conjunction with BRCA1-CtIP Complexes. Nucleic Acids Res..

[47] Kraft D., Rall M., Volcic M., Metzler E., Groo A., Stahl A., Bauer L., Nasonova E., Salles D., Taucher-Scholz G. (2015). NF-KappaB-Dependent DNA Damage-Signaling Differentially Regulates DNA Double-Strand Break Repair Mechanisms in Immature and Mature Human Hematopoietic Cells. Leukemia.

[48] Zhang Y., Maria-Villanueva M., Krieger S., Ramesh G.T., Neelam S., Wu H. (2017). Transcriptomics, NF-ΚB Pathway, and Their Potential Spaceflight-Related Health Consequences. Int. J. Mol. Sci..

[49] He Y., Pasupala N., Zhi H., Dorjbal B., Hussain I., Shih H.M., Bhattacharyya S., Biswas R., Miljkovic M., Semmes O.J. (2021). NF-ΚB–Induced R-Loop Accumulation and DNA Damage Select for Nucleotide Excision Repair Deficiencies in Adult T Cell Leukemia. Proc. Natl. Acad. Sci. USA.

[50] Ragu S., Droin N., Matos-Rodrigues G., Barascu A., Caillat S., Zarkovic G., Siberchicot C., Dardillac E., Gelot C., Guirouilh-Barbat J. (2023). A Noncanonical Response to Replication Stress Protects Genome Stability through ROS Production, in an Adaptive Manner. Cell Death Differ..

[51] Rithidech K.N., Honikel L., Whorton E.B. (2007). MFISH Analysis of Chromosomal Damage in Bone Marrow Cells Collected from CBA/CaJ Mice Following Whole Body Exposure to Heavy Ions (56Fe Ions). Radiat. Environ. Biophys..

[52] Singh R., Rajput M., Singh R.P. (2021). Simulated Microgravity Triggers DNA Damage and Mitochondria-Mediated Apoptosis through ROS Generation in Human Promyelocytic Leukemic Cells. Mitochondrion.

[53] Löbrich M., Jeggo P. (2017). A Process of Resection-Dependent Nonhomologous End Joining Involving the Goddess Artemis. Trends Biochem. Sci..

[54] Bartosova Z., Krejci L. (2014). Nucleases in Homologous Recombination as Targets for Cancer Therapy. FEBS Lett..

[55] Trenner A., Sartori A.A. (2019). Harnessing DNA Double-Strand Break Repair for Cancer Treatment. Front. Oncol..

[56] Murina O., von Aesch C., Karakus U., Ferretti L.P., Bolck H.A., Hänggi K., Sartori A.A. (2014). FANCD2 and CtIP Cooperate to Repair DNA Interstrand Crosslinks. Cell Rep..

[57] Claesson K., Magnander K., Kahu H., Lindegren S., Hultborn R., Elmroth K. (2011). RBE of α-Particles from 211At for Complex DNA Damage and Cell Survival in Relation to Cell Cycle Position. Int. J. Radiat. Biol..

[58] Pinto M., Prise K.M., Michael B.D. (2005). Evidence for Complexity at the Nanometer Scale of Radiation-Induced DNA DSBs as a Determinant of Rejoining Kinetics. Radiat. Res..

[59] Anderson J.A., Harper J.V., Cucinotta F.A., O’Neill P. (2010). Participation of DNA-PKcs in DSB Repair after Exposure to High-and Low-Let Radiation. Radiat. Res..

[60] Nickoloff J.A., Sharma N., Taylor L. (2020). Clustered DNA Double-Strand Breaks: Biological Effects and Relevance to Cancer Radiotherapy. Genes..

[61] Panier S., Boulton S.J. (2014). Double-Strand Break Repair: 53BP1 Comes into Focus. Nat. Rev. Mol. Cell Biol..

[62] Becker D., Elsasser T., Tonn T., Seifried E., Durante M., Ritter S., Fournier C. (2009). Response of Human Hematopoietic Stem and Progenitor Cells to Energetic Carbon Ions. Int. J. Radiat. Biol..

[63] Ritter S., Durante M. (2010). Heavy-Ion Induced Chromosomal Aberrations: A Review. Mutat. Res..

[64] Iliakis G., Mladenov E., Mladenova V. (2019). Necessities in the Processing of DNA Double Strand Breaks and Their Effects on Genomic Instability and Cancer. Cancers.

[65] Cornforth M.N. (2021). Occam’s Broom and the Dirty DSB: Cytogenetic Perspectives on Cellular Response to Changes in Track Structure and Ionization Density. Int. J. Radiat. Biol..

[66] Mangala L.S., Zhang Y., He Z., Emami K., Ramesh G.T., Story M., Rohde L.H., Wu H. (2011). Effects of Simulated Microgravity on Expression Profile of MicroRNA in Human Lymphoblastoid Cells. J. Biol. Chem..

[67] Zhang Y., Lu T., Wong M., Wang X., Stodieck L., Karouia F., Story M., Wu H. (2016). Transient Gene and MicroRNA Expression Profile Changes of Confluent Human Fibroblast Cells in Spaceflight. FASEB J..

[68] Zwart S.R., Pierson D., Mehta S., Gonda S., Smith S.M. (2010). Capacity of Omega-3 Fatty Acids or Eicosapentaenoic Acid to Counteract Weightlessness-Induced Bone Loss by Inhibiting NF-ΚB Activation: From Cells to Bed Rest to Astronauts. J. Bone Miner. Res..

[69] Chang T.T., Walther I., Li C.-F., Boonyaratanakornkit J., Galleri G., Meloni M.A., Pippia P., Cogoli A., Hughes-Fulford M. (2012). The Rel/NF-ΚB Pathway and Transcription of Immediate Early Genes in T Cell Activation Are Inhibited by Microgravity. J. Leukoc. Biol..

[70] Boonyaratanakornkit J.B., Cogoli A., Li C.-F., Schopper T., Pippia P., Galleri G., Meloni M.A., Hughes-Fulford M. (2005). Key Gravity-sensitive Signaling Pathways Drive T-cell Activation. FASEB J..

[71] Paulsen K., Thiel C., Timm J., Schmidt P.M., Huber K., Tauber S., Hemmersbach R., Seibt D., Kroll H., Grote K.H. (2010). Microgravity-Induced Alterations in Signal Transduction in Cells of the Immune System. Acta Astronaut..

[72] Horneck G., Rettberg P., Kozubek S., Baumstark-Khan C., Rink H., Schäfer M., Schmitz C. (1997). The Influence of Microgravity on Repair of Radiation-Induced DNA Damage in Bacteria and Human Fibroblasts. Radiat. Res..

[73] Berti M., Cortez D., Lopes M. (2020). The Plasticity of DNA Replication Forks in Response to Clinically Relevant Genotoxic Stress. Nat. Rev. Mol. Cell Biol..

[74] Ihle M., Biber S., Schroeder I.S., Blattner C., Deniz M., Damia G., Gottifredi V., Wiesmuller L. (2021). Impact of the Interplay between Stemness Features, P53 and Pol Iota on Replication Pathway Choices. Nucleic Acids Res..

[75] Schlacher K., Wu H., Jasin M. (2012). A Distinct Replication Fork Protection Pathway Connects Fanconi Anemia Tumor Suppressors to RAD51-BRCA1/2. Cancer Cell.

[76] Petropoulos M., Champeris Tsaniras S., Taraviras S., Lygerou Z. (2019). Replication Licensing Aberrations, Replication Stress, and Genomic Instability. Trends Biochem. Sci..

[77] Zhao T., Li R., Tan X., Zhang J., Fan C., Zhao Q., Deng Y., Xu A., Lukong K.E., Genth H. (2018). Simulated Microgravity Reduces Focal Adhesions and Alters Cytoskeleton and Nuclear Positioning Leading to Enhanced Apoptosis via Suppressing FAK/Rhoa-Mediated MTORC1/NF-ΚB and ERK1/2 Pathways. Int. J. Mol. Sci..

[78] Mahen R., Hattori H., Lee M., Sharma P., Jeyasekharan A.D., Venkitaraman A.R. (2013). A-Type Lamins Maintain the Positional Stability of DNA Damage Repair Foci in Mammalian Nuclei. PLoS ONE.

[79] Simon M., Yang J., Gigas J., Earley E.J., Hillpot E., Zhang L., Zagorulya M., Tombline G., Gilbert M., Yuen S.L. (2022). A Rare Human Centenarian Variant of SIRT6 Enhances Genome Stability and Interaction with Lamin A. EMBO J..

[80] Barravecchia I., De Cesari C., Forcato M., Scebba F., Pyankova O.V., Bridger J.M., Foster H.A., Signore G., Borghini A., Andreassi M. (2022). Microgravity and Space Radiation Inhibit Autophagy in Human Capillary Endothelial Cells, through Either Opposite or Synergistic Effects on Specific Molecular Pathways. Cell. Mol. Life Sci..

[81] Schreck R., Rieber P., Baeuerle P.A. (1991). Reactive Oxygen Intermediates as Apparently Widely Used Messengers in the Activation of the NF-ΚB Transcription Factor and HIV-1. EMBO J..

[82] Albi E., Krüger M., Hemmersbach R., Lazzarini A., Cataldi S., Codini M., Beccari T., Ambesi-Impiombato F.S., Curcio F. (2017). Impact of Gravity on Thyroid Cells. Int. J. Mol. Sci..

[83] Eiermann P., Kopp S., Hauslage J., Hemmersbach R., Gerzer R., Ivanova K. (2013). Adaptation of a 2-D Clinostat for Simulated Microgravity Experiments with Adherent Cells. Microgravity Sci. Technol..

[84] Kamentsky L., Jones T.R., Fraser A., Bray M.-A., Logan D.J., Madden K.L., Ljosa V., Rueden C., Eliceiri K.W., Carpenter A.E. (2011). Improved Structure, Function and Compatibility for CellProfiler: Modular High-Throughput Image Analysis Software. Bioinformatics.

[85] Fenech M. (2007). Cytokinesis-Block Micronucleus Cytome Assay. Nat. Protoc..

[86] Rall-Scharpf M., Friedl T.W.P., Biechonski S., Denkinger M., Milyavsky M., Wiesmüller L. (2021). Sex-Specific Differences in DNA Double-Strand Break Repair of Cycling Human Lymphocytes during Aging. Aging.

[87] Park E., Kim H., Kim J.M., Primack B., Vidal-Cardenas S., Xu Y., Price B.D., Mills A.A., D’Andrea A.D. (2013). FANCD2 Activates Transcription of TAp63 and Suppresses Tumorigenesis. Mol. Cell.

[88] Helbling-Leclerc A., Dessarps-Freichey F., Evrard C., Rosselli F. (2019). Fanconi Anemia Proteins Counteract the Implementation of the Oncogene-Induced Senescence Program. Sci. Rep..

[89] Flach J., Bakker S.T., Mohrin M., Conroy P.C., Pietras E.M., Reynaud D., Alvarez S., Diolaiti M.E., Ugarte F., Forsberg E.C. (2014). Replication Stress Is a Potent Driver of Functional Decline in Ageing Haematopoietic Stem Cells. Nature.

[90] Walter D., Lier A., Geiselhart A., Thalheimer F.B., Huntscha S., Sobotta M.C., Moehrle B., Brocks D., Bayindir I., Kaschutnig P. (2015). Exit from Dormancy Provokes DNA-Damage-Induced Attrition in Haematopoietic Stem Cells. Nature.

[91] Sotnezova E.V., Markina E.A., Andreeva E.R., Buravkova L.B. (2017). Myeloid Precursors in the Bone Marrow of Mice after a 30-Day Space Mission on a Bion-M1 Biosatellite. Bull. Exp. Biol. Med..

[92] Mao X.W., Boerma M., Rodriguez D., Campbell-Beachler M., Jones T., Stanbouly S., Sridharan V., Nishiyama N.C., Wroe A., Nelson G.A. (2019). Combined Effects of Low-Dose Proton Radiation and Simulated Microgravity on the Mouse Retina and the Hematopoietic System. Radiat. Res..

