# Supplementary material for: Simulating Space Conditions Evokes Different DNA Damage Responses in Immature and Mature Cells of the Human Hematopoietic System
Source: Int J Mol Sci. 2023 Sep 6;24(18):13761. doi: 10.3390/ijms241813761 (PMC10531023; doi:10.3390/ijms241813761)
Supplement: Supplementary file 1 [file ijms-24-13761-s001.zip › ijms-2546388-supplementary.pdf]

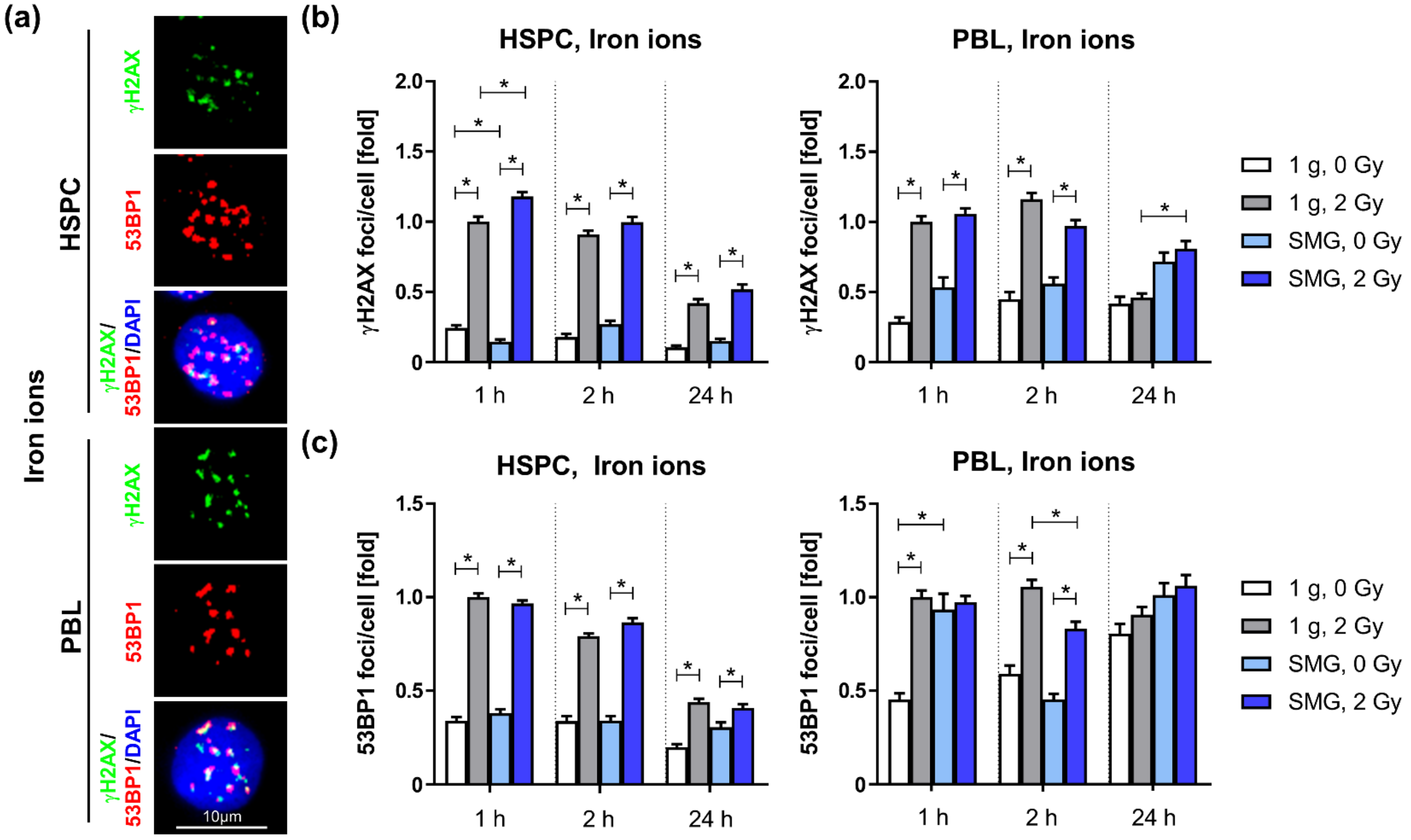

Figure S1: Repair of Iron ion-induced DNA damage in simulated microgravity (SMG)
HSPC and PBL were cultured under SMG or normal gravity (1 g) conditions for 24 h before they were exposed to a dose of 2 Gy Iron ions (150 keV/µm) and further cultivated. At indicated time points cells were fixed, immunolabeled and analyzed by immunofluorescence microscopy. **(a) Exemplary immunofluorescence images** of nuclei with γH2AX and 53BP1 foci (1 g, 2 Gy, 1 h). **(b) γH2AX and (c) 53BP1 foci**. Foci numbers of 47-369 nuclei were scored for each condition and at each timepoint per experiment (N). Columns represent relative mean foci numbers; bars, SEM; Mann-Whitney; *, $p<0.0001$; HSPC: N=3, PBL N=2. 1 relative focus represents the following mean scores for HSPC: γH2AX: 8 foci/cell, 53BP1: 9 foci/cell, PBL: γH2AX: 10 foci/cell, 53BP1: 9 foci/cell.

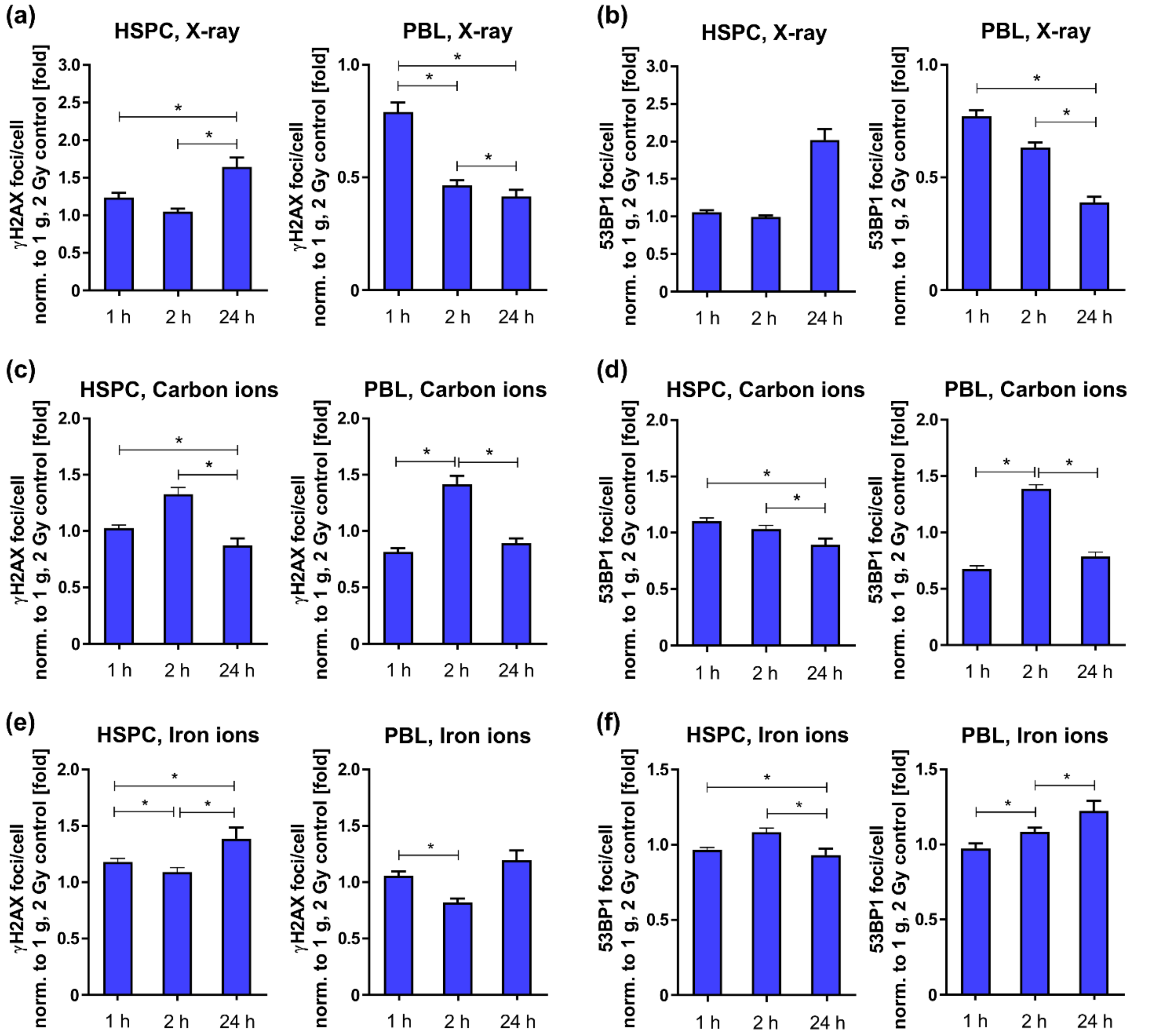

Figure S2: Radiation-induced DNA damage foci numbers under SMG corrected for 1 g controls

Mean γH2AX (a, c, e) and 53BP1 (b, d, f) foci numbers of HSPC and PBL exposed to radiation and SMG (presented in Fig. 2 and Fig. S1) were normalized to the irradiated 1 g control for each time point. HSPC and PBL were cultured under SMG conditions for 24 h before they were exposed to a dose of 2 Gy X-rays **(a, b),** Carbon ions (75 keV/μm) **(c, d)** or Iron ions (150 keV/μm) **(e, f)** and further cultivated. At indicated time points cells were fixed, immunolabeled and analyzed by immunofluorescence microscopy.

X-ray-induced γH2AX (a) and 53BP1 foci (b) under SMG normalized to 1 g controls (N=3). **Carbon ion-induced γH2AX (c) and 53BP1 foci (d) under SMG normalized to 1 g controls** (N=4). **Iron ion-induced γH2AX (e) and 53BP1 (f) foci under SMG normalized to 1 g controls** (HSPC: N=3, PBL: N=2). Columns represent relative mean foci numbers; bars, SEM; Mann-Whitney; *, $p<0.0001$.

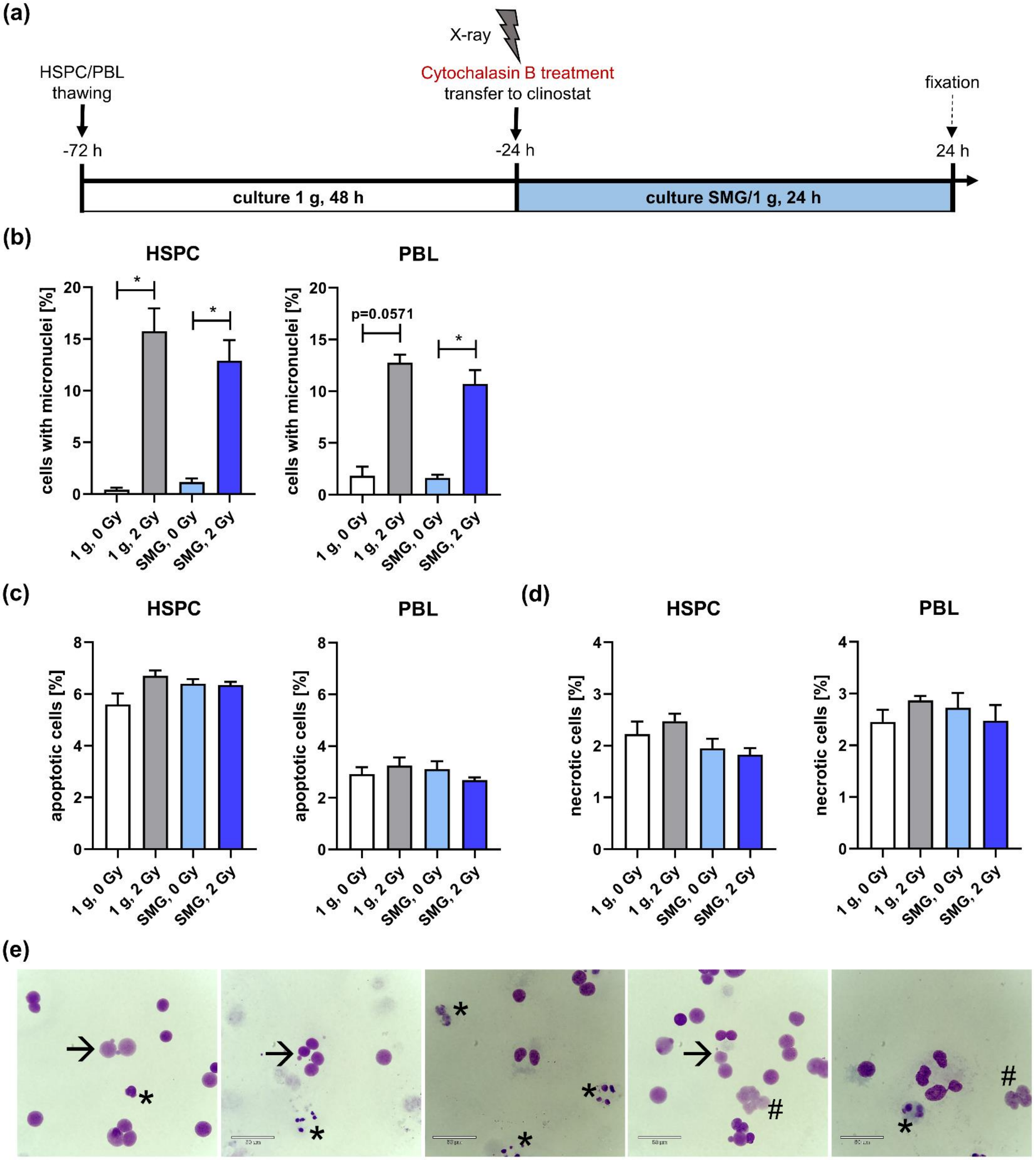

Figure S3: Influence of simulated micrograviy (SMG) on genomic stability

(a) Schematic overview of the cell culture set-up. HSPC and PBL were thawed and cultured in a cell culture flask under normal gravity (1 g) for 48 h (white bar). Cells were then treated with Cytocalasin B and exposed to a dose of 2 Gy X-rays before they were further cultured in the clinostat or sham clinostat under SMG or 1 g conditions respectively for 48 h (blue bar). Then cells were fixed, Giemsa stained and microscopically analyzed. **(b)** Percentage of **cells with micronuclei** within the binucleated cells. **(c, d) Percentage of apoptotic and necrotic cells** within the whole cell population. (b-d) Columns, mean cell count, bars, SEM; Mann-Whitney; *, $p<0.05$; N=1, n=3-4. **(e) Exemplary images of Giemsa-stained PBL.** Giemsa staining allows identification of cells with micronuclei (→) as well as apoptotic (*) or necrotic cells (#).

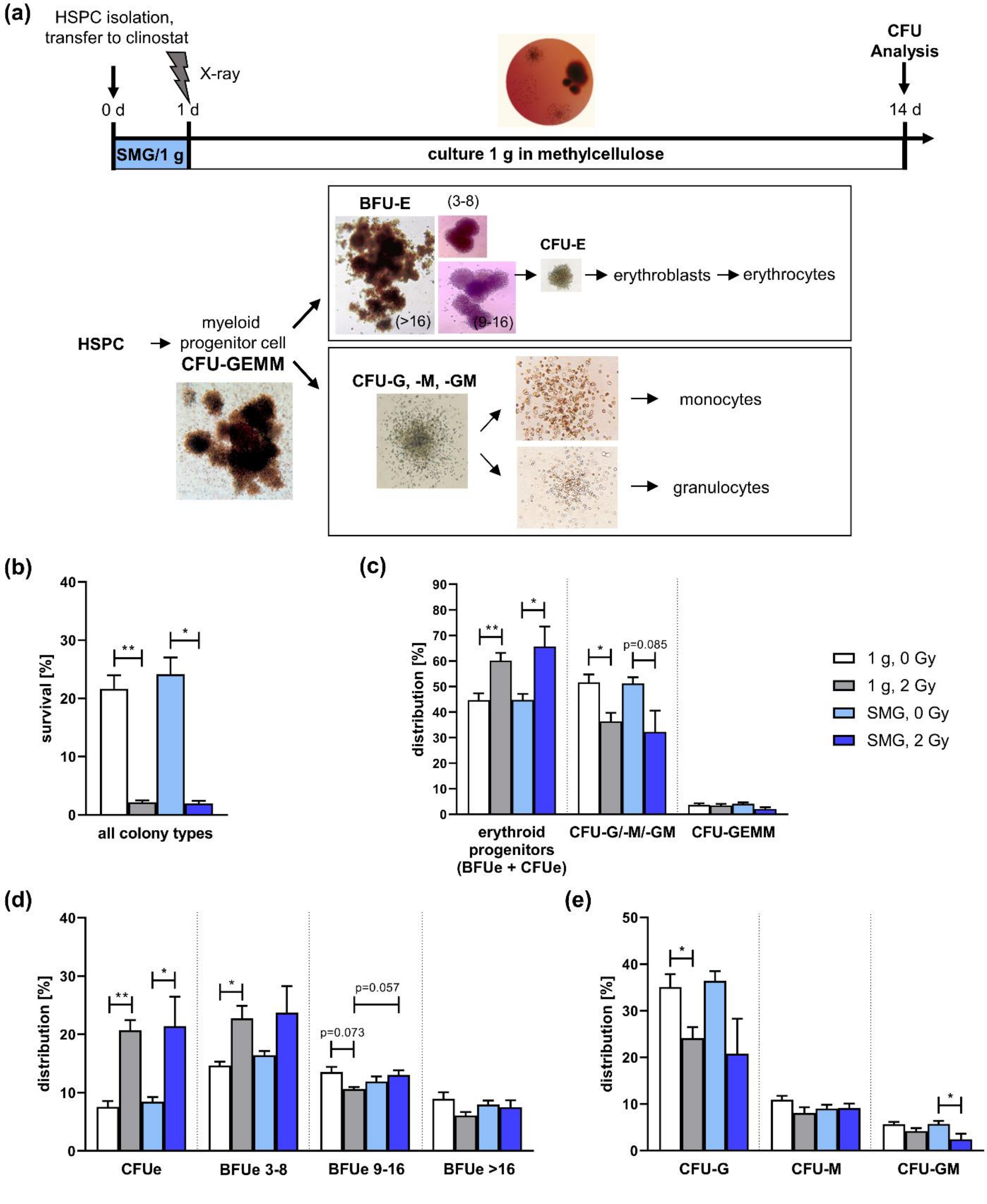

Figure S4: Influence of simulated microgravity (SMG) on HSPC differentiation

(a) Schematic overview of cell culture set-up for colony forming units (CFU) assays (b-e). Freshly isolated HSPC were either cultured under SMG or 1 g for 24 h before they were irradiated with a dose of 2 Gy X-rays and seeded in semisolid methylcellulose. After 14 days of growth at 1 g colonies were determined based on morphological pattern. **(b-e) Growth and distribution of myeloid colonies.** Sum of all colony types = 100%. At least 2 up to 6 samples have been analyzed per experiment (N). Columns represent mean; bars SEM; Mann-Whitney; *, $p<0.05$; **, $p<0.01$; 1 g, 0 Gy, N=8; SMG, 0 Gy, N=8; 1 g 2 Gy, N=4; SMG 2 Gy, N=3. **(b) Survival of all colony forming units** (including all colony types), percentage was calculated based on the number of originally seeded cells. **(c) Distribution of different colony types**: colonies derived from erythroid progenitors, including later-stage colony-forming unit-erythroid (CFU-e, 1-2 clusters) and more primitive burst-forming unit-erythroid with high proliferative capacity (BFU-e, 3-8, 9-19, >16 clusters), granulocyte/macrophage progenitors, including colony-forming unit-granulocyte, (CFU-G), colony-forming unit-macrophage (CFU-M) and colony-forming unit-granulocyte, macrophage (CFU-GM) and colony forming unit-granulocyte, erythroid, macrophage, megakaryocyte (CFU-GEMM) derived from multi-potential progenitors. **(d) Distribution of erythroid colonies. (e) Distribution of granulocyte/macrophage colonies.**

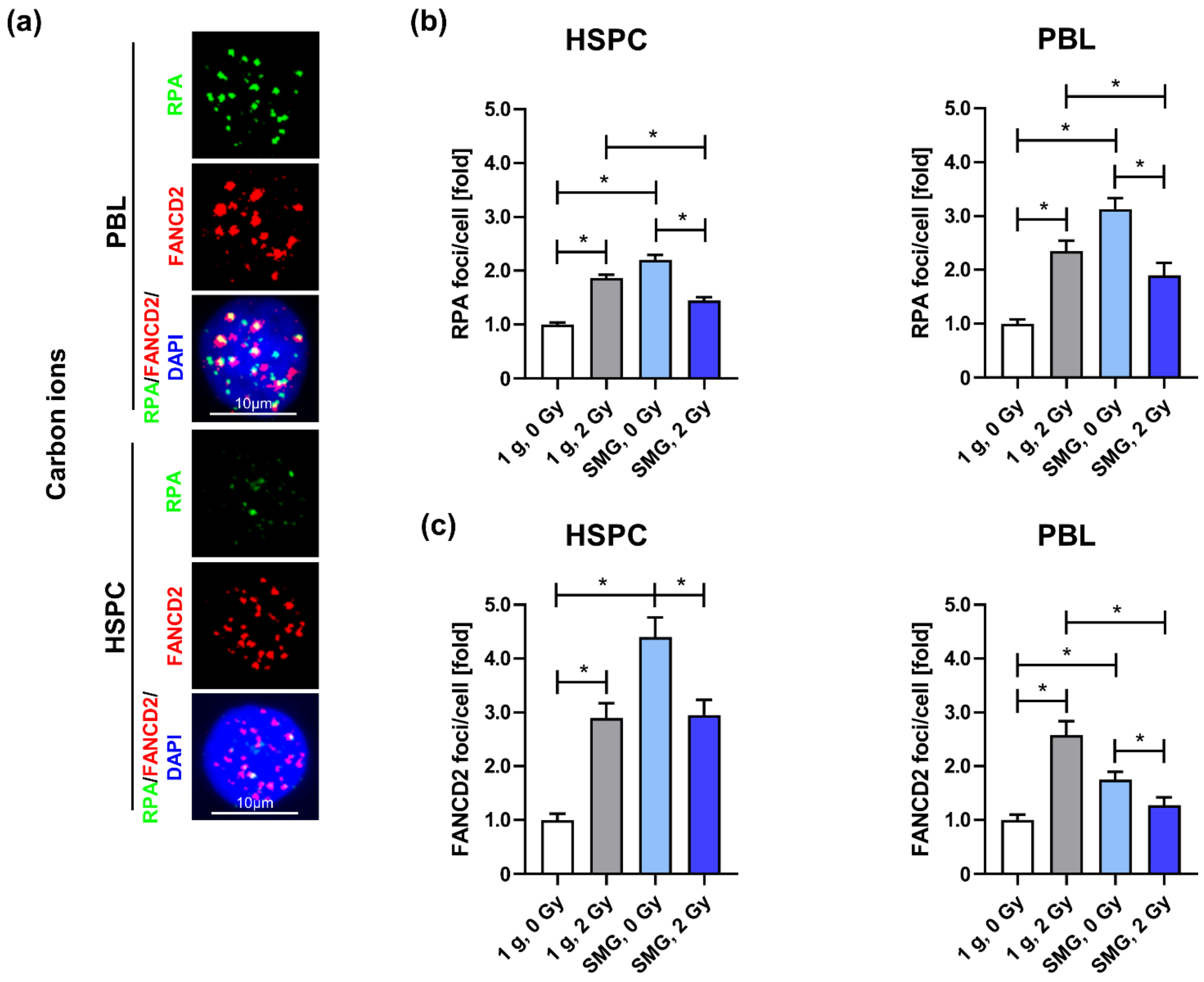

Figure S5: Homology-mediated repair of Carbon ion-induced DNA damage in simulated microgravity (SMG) HSPC and PBL were cultured under SMG or normal gravity (1 g) conditions for 24 h before they were exposed to a dose of 2 Gy Carbon ions (75 keV/µm) and further cultivated. 2 h post irradiation cells were fixed, immunolabeled and analyzed by immunofluorescence microscopy. **(a) Exemplary immunofluorescence images** of nuclei with RPA and FANCD2 foci (1 g, 2 Gy, 2 h). **(b) RPA and (c) FANCD2 foci**. Foci numbers of 36-186 nuclei were scored for each condition per experiment. Columns represent relative mean foci numbers; bars, SEM; Mann-Whitney; *, p<0.0001; N=2 (HSPC); N=3 (PBL). 1 relative focus represents the following mean scores for HSPC: RPA: 10 foci/cell and FANCD2: 3 foci/cell and PBL: RPA: 2 foci/cell and FANCD2: 1 foci/cell.

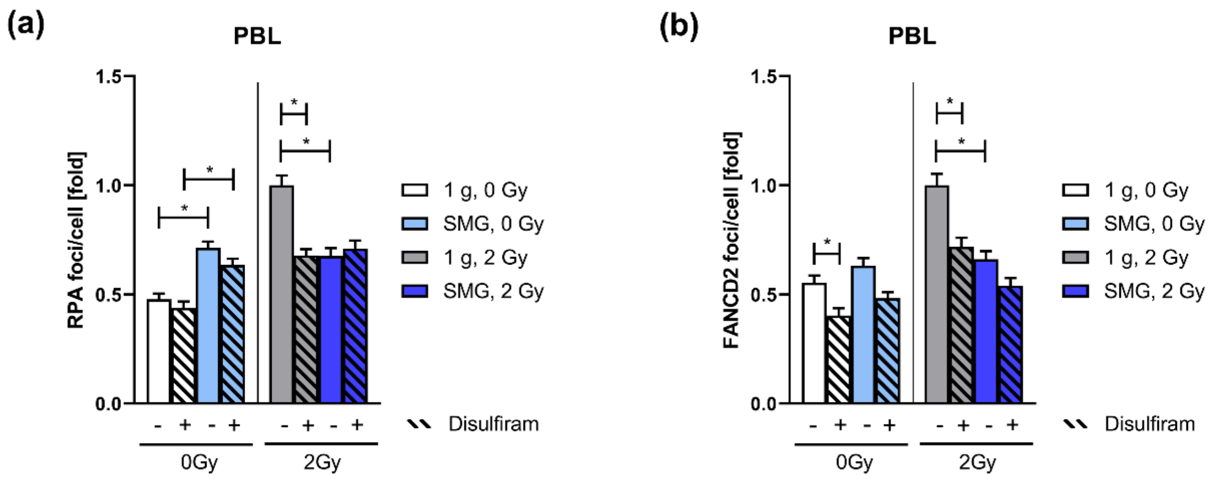

Figure S6: Role of NF-κB signaling in homology-directed repair in simulated microgravity (SMG)
PBL were thawed and cultured in a cell culture flask under normal gravity (1 g) for 48 h before they were cultured in the clinostat or sham clinostat under SMG or 1 g conditions respectively for 48 h. After 20 h (-4 h) cells were treated with the NF-κB-inhibitor Disulfiram (Dis) (4 µM) or mock-treated with DMSO before they were exposed to a dose of 2 Gy X-rays and further cultivated. 2 h post radiation exposure cells were fixed, immunolabeled and analyzed by immunofluorescence microscopy. **RPA (a) and FANCD2 (b) foci.** Foci numbers of 36-186 nuclei were scored for each condition and at each time point per experiment. Columns represent mean foci numbers; bars, SEM; Mann-Whitney; *, $p<0.0001$; N=3. 1 g, 2 Gy, DMSO was set to 1. 1 relative focus represents the following mean scores RPA: 31 foci/cell and FANCD2: 26 foci/cell.
